# Antidepressant class and concurrent rTMS outcomes in major depressive disorder: a systematic review and meta-analysis

**DOI:** 10.1016/j.eclinm.2024.102760

**Published:** 2024-07-27

**Authors:** Alina Zaidi, Rafeya Shami, Isabella J. Sewell, Xingshan Cao, Peter Giacobbe, Jennifer S. Rabin, Maged Goubran, Clement Hamani, Walter Swardfager, Benjamin Davidson, Nir Lipsman, Sean M. Nestor

**Affiliations:** a Hurvitz Brain Sciences Program, Sunnybrook Research Institute, University of Toronto, Toronto, ON, Canada; b Harquail Centre for Neuromodulation, Sunnybrook Health Sciences Centre, Toronto, ON, Canada; c Institute of Medical Science, University of Toronto, Toronto, ON, Canada; d Research Design and Biostatistics, Sunnybrook Research Institute, Toronto, ON, Canada; e Department of Psychiatry, Temerty Faculty of Medicine, University of Toronto, Toronto, ON, Canada; f Department of Neurology, Temerty Faculty of Medicine, University of Toronto, Toronto, ON, Canada; g Department of Neurosurgery, Temerty Faculty of Medicine, University of Toronto, Toronto, ON, Canada; h Department of Pharmacology & Toxicology, University of Toronto, Toronto, ON, Canada; i Rehabilitation Sciences Institute, University of Toronto, Toronto, ON, Canada; j Department of Medical Biophysics, University of Toronto, Toronto, ON, Canada

**Keywords:** Transcranial magnetic stimulation, Antidepressant, Major depressive disorder, Selective serotonin reuptake inhibitors, Serotonin norepinephrine reuptake inhibitors

## Abstract

**Background:**

Repetitive transcranial magnetic stimulation (rTMS) is frequently used as an adjunctive treatment with antidepressants for depression. We aimed to evaluate the clinical efficacy and safety of antidepressant classes when administered concurrently with rTMS for the management of major depressive disorder (MDD).

**Methods:**

In this systematic review and meta-analysis, MEDLINE, Embase, PsycINFO, and the Cochrane Library were searched from inception to April 12th 2024 for terms relating to medication, depression, and rTMS and appraised by 2 independent screeners. All randomized clinical trials that prospectively evaluated a specific antidepressant adjunctively with sham rTMS as a control in MDD were included. The study was registered with PROSPERO (CRD42023418435). The primary outcome measure assessed symptomatic improvement measured by formal depression scales. We used a random-effects model with pooled Standardized Mean Differences (SMDs) and log odds ratios (OR). All studies were assessed for their methodological quality and bias using the Cochrane Collaboration Risk of Bias tool version 2 (RoB2).

**Findings:**

14 articles from 5376 identified studies were included in the systematic review and meta-analysis. There was only sufficient trial data to evaluate the effects of rTMS and combination therapy with selective serotonin reuptake inhibitors (SSRIs) and selective norepinephrine reuptake inhibitors (SNRIs). Across studies, 848 participants (mean [SD] age:41.1 [18.7] years for SSRIs, 51.8 [3.8] years for SNRIs) prospectively examined the efficacy of antidepressant medication with rTMS. Combining rTMS with SSRIs led to significantly lower depression scores, (SMD [CI] of −0.65 [−0.98, −0.31], p = 0.0002, I^2^ = 66.1%), higher response (OR = 0.97 [0.50, 1.44], p < 0.0001, I^2^ = 25.33%) and remission rates (OR = 1.04 [0.55, 1.52], p < 0.0001, I^2^ = 0.00%) than medication with sham rTMS. No additive benefit was found for SNRIs with rTMS (SMD of 0.10 [−0.14, 0.34], p = 0.42, I^2^ = 0.00%; OR = 0.12 [−0.39, 0.62], p = 0.64, I^2^ = 0.00%; OR = −0.31 [−0.90, 0.28], p = 0.86, I^2^ = 39.9%). The overall risk of bias for the included studies ranged from low to high, with 1 study having a high risk of bias.

**Interpretation:**

The combination of rTMS with SSRIs, but not SNRIs, significantly reduced depression severity, increasing response and remission rates. Some analyses demonstrated high heterogeneity, which was influenced by an SSRI trial with a high effect size. Overall, these results suggest that not all antidepressant combination therapies are alike, and SSRIs should be considered when initiating rTMS.

**Funding:**

Donald T. Stuss Young Investigator Research Innovation Award from the Sandra Black Centre for Brain Resilience & Recovery and the 10.13039/100031205Harquail Centre for Neuromodulation through the 10.13039/100019746Sunnybrook Foundation.

**Research in context**

**Evidence before this study**

MEDLINE, Embase, and PsychINFO were searched from inception to April 2024 for terms relating to medication, depression, and repetitive transcranial magnetic stimulation (rTMS) (full search strategy and terms are included in Supplementary Methods S1–S3). Randomized clinical trials have demonstrated superior clinical outcomes when antidepressant medications were initiated with acute rTMS therapy in major depressive disorder (MDD). Previous meta-analyses reported that antidepressants commenced with rTMS demonstrated greater efficacy than antidepressants with sham rTMS. There were no reviews of randomized, sham-controlled trials evaluating class-specific effects of antidepressants and adjunctive rTMS.

**Added value of this study**

To our knowledge, this was the first systematic review and meta-analysis evaluating the effect of antidepressant class when initiated prospectively with acute rTMS versus with sham rTMS treatment in MDD. We show that selective serotonin reuptake inhibitors (SSRIs), but not Serotonin Norepinephrine Reuptake inhibitors (SNRIs), improved clinical efficacy when concurrently started with acute rTMS. Secondary analyses revealed that neither treatment duration nor dose affected clinical outcomes, suggesting that low doses of SSRIs initiated concurrently with a shorter duration of rTMS treatments may be equally beneficial to patients.

**Implications of all the available evidence**

Our findings suggest a need to consider antidepressants, and specifically the class of medication, when starting adjunctive acute neuromodulation therapies. Research should be prioritized to prospectively evaluate other classes of antidepressants against placebo in combination with acute rTMS treatments. Clinics can use these findings to optimize patient care by commencing an SSRI with acute rTMS to enhance antidepressant outcomes across the spectrum of treatment-resistant MDD. In addition, fewer rTMS treatment sessions and lower SSRI doses may improve tolerability while improving clinical efficacy.

## Introduction

Antidepressants are effective for managing major depressive disorder (MDD) symptoms of moderate-to-severe intensity, yet 50–60% of individuals do not tolerate or adequately respond to first-line antidepressant therapy, and some may receive repetitive transcranial magnetic stimulation (rTMS). rTMS is a non-invasive brain stimulation technique that uses a strong electromagnetic field to induce changes to networks subserving emotion, cognition and behaviour.^1^

Although rTMS is an established treatment for moderate-to-severe treatment-resistant depression, relapse rates are up to 70% following an acute treatment series and clinical response varies greatly between protocols.^2^, ^3^, ^4^ Most modern rTMS trials enrolled patients who failed first-line antidepressants or were continued on partially ineffective medication during rTMS.^5^ Relapse-prevention strategies following rTMS are unclear and involve various rTMS continuation protocols, pharmacotherapy, and psychotherapy. Antidepressants such as selective serotonin reuptake inhibitors (SSRIs), and serotonin and norepinephrine reuptake inhibitors (SNRIs) are established maintenance treatments for MDD that can reduce relapse rates up to 50%, including after acute rTMS.^6^, ^7^, ^8^ Randomized clinical trials and meta-analyses have demonstrated superior clinical response when antidepressants were initiated with acute rTMS.^9^ This relationship has also been observed across the lifespan^10^, ^11^, ^12^ and when comorbid illness, such as multiple sclerosis and stroke, is present.^13^, ^14^, ^15^, ^16^ However, tremendous heterogeneity exists among antidepressant mechanisms, and an evidence-based choice of medication to initiate concurrently with rTMS has not been evaluated by meta-analysis.

The aim of this systematic review was to compare the efficacy of antidepressant classes when initiated concurrently with acute rTMS for the treatment of a major depressive episode. We performed a systematic review and meta-analysis of all randomized, sham-controlled trials that prospectively examined combination antidepressant therapy with acute rTMS protocols in MDD. We also analysed the pooled effects across all antidepressants when started concurrently with rTMS. Changes in formal clinical depression scale scores were examined as primary outcome measures. Response rates, remission rates, and safety data was also assessed across antidepressant classes when administered with rTMS.

## Methods

### Search strategy and selection criteria

This review and meta-analysis were registered *a priori* with the Prospero database [CRD42023418435] and followed the Preferred Reporting Items for Systematic Review (PRISMA) guidelines.^17^ Relevant references were identified through systematic searches of MEDLINE, Embase, PsycINFO, and the Cochrane Library from inception to April 12th, 2024. Additional sources were identified through forward and backward citation searches in Google Scholar. Databases were searched using combinations of the following search terms: ‘major depressive disorder’, ‘MDD’, ‘depression’, ‘pharmacotherapy’, ‘pharmacology’, ‘medication’, ‘psychotropic’, ‘drug therapy’, ‘psychopharmaceutical’, ‘SSRI’, ‘selective serotonin reuptake inhibitors’, ‘SNRI’, ‘serotonin and norepinephrine reuptake inhibitors’, ‘TCA’, ‘tricyclic antidepressants’, ‘MAOI’, ‘monoamine oxidase inhibitors’, ‘benzodiazepines’, ‘atypical antidepressants’, ‘typical antipsychotics’, ‘atypical antipsychotics’, ‘stimulants’, ‘psychostimulants’, ‘repetitive transcranial magnetic stimulation’, ‘transcranial magnetic stimulation’, ‘TMS’, ‘rTMS’, ‘TBS’, ‘Theta Burst Stimulation’, ‘iTBS’, ‘deep transcranial magnetic stimulation’, ‘dTMS’. The full search strategy is included in the supplement (Supplementary Methods S1–S3).

Inclusion and exclusion criteria for the study population, intervention, comparators, controls, and study design were established prior to screening articles. Inclusion criteria followed the PICOS framework and were as follows: 1) Patients with a current diagnosis of MDD; 2) Patients who underwent an acute rTMS treatment protocol (all therapeutic rTMS protocols were included) while concurrently initiating a specific antidepressant medication; 3) Studies with a sham-rTMS control arm; 4) The primary outcomes assessed clinical efficacy as measured by formal depression scales (i.e., change in depression severity, response, or remission) 5) Prospective, randomized controlled trials reported in English. Exclusion criteria were: 1) Patients with other mental disorders (including bipolar depression); 3) Conference abstracts, case studies, reviews, retrospective chart studies; 4) Studies not reported in English.

All studies were independently screened by two reviewers (AZ & RS) using Covidence (Veritas Health Innovation, Melbourne). Articles identified as relevant by both reviewers were retrieved, and full texts were screened. Articles independently selected by both reviewers after the full-text screening were included in the final review. Any discrepancies between reviewers were resolved by a third reviewer (SN).

### Data extraction

Data was extracted by 2 independent reviewers (AZ & RS) and was determined *a priori* and included author(s), publication year, country, study design, sample size, mean age of sample, sex reported as % female, primary diagnosis, rTMS protocol, psychotropic medication, treatment arms, formal depression scale measures, remission rate, response rate, and safety/tolerability data. Treatment resistant depression is formally defined as failing 2 or more antidepressant trials, however due to the paucity of information related to the number of prior antidepressant trials, other data related to treatment to resistance was extracted such as duration of illness/depressive episode, number of prior depressive episodes, and failed treatments to gain further insight into the variability in depression severity between study populations.

### Outcomes

The primary outcome variable was change in depression symptomatology from baseline to endpoint as measured by a formal depression scale such as the Montgomery-Åsberg Depression Rating Scale (MADRS), Hamilton Depression Rating Scale (HAMD), or Beck Depression Inventory (BDI). We used the formal scale that was used as the primary outcome measure for a given study. We considered the first depression score measured immediately prior to treatment the baseline. The endpoint depression score was the first depression scale measured immediately post-treatment. Secondary outcome measures included response and remission rates as measured by standardized depression scales. A response was defined as a 50% reduction or greater from the baseline depression scale score. The remission rate was defined per study based on established cut-off scores for formal depression scales. Additionally, we performed meta-analyses to compare efficacy outcomes between patients receiving active treatment with rTMS and medication (treatment group) compared to sham rTMS and medication (control group). Safety and tolerability outcomes were examined across studies, using the number of reported dropouts during the treatment phase and incidence of serious and other adverse events.

### Statistics

Standardized mean differences (SMDs) were calculated for continuous outcome measures (e.g., depression severity). SMDs were calculated as the difference between patients receiving active rTMS and medication (treatment group) versus those receiving sham rTMS and medication (control group). Pooling of SMDs across studies was performed using a random-effects model and the DerSimonian and Laird method.^18^ For the primary outcome measure, a negative SMD indicated that the treatment group had a greater decline in depression severity than the control group. For our primary analysis, we stratified by outcome and major chemical psychotropic class (e.g., SSRI, SNRI, etc.). For dichotomous outcomes (e.g., remission rate and response rate), log odds ratios (OR) were calculated and then pooled across studies per psychotropic class using the Mantel-Haenszel fixed-effects model. We decided to use log OR over risk ratios to maintain a symmetrical outcome definition. A positive OR indicated that the treatment group had a greater quantity for an outcome of interest (response/remission) than the control group. To confirm the main findings, sensitivity analyses were performed for the primary and secondary endpoints by removing studies that reported less treatment-resistant samples or patients treated for a first major depressive episode to account for sampling differences across study populations. A subgroup analysis explored the effect of specific medications on depression severity as well as treatment duration (weeks), and a meta-regression was performed to assess the impact of dose in fluoxetine equivalent and number of rTMS treatment sessions.^19^ We additionally pooled all antidepressants to assess the global effect of antidepressants with rTMS. Heterogeneity across studies for all meta-analyses was assessed using the Cochran’s Q test (statistical significance set at p < .10) and I^2^ statistics.^20^^,^^21^ All statistical analyses were performed using the Metafor package in R version 4.0.3 (Foundation for Statistical Computing, Vienna, Austria). Prospective randomized studies were assessed for their methodological quality and any bias using the Cochrane Collaboration Risk of Bias tool version 2 (RoB2).^22^ The RoB2 tool assesses studies using the following domains: (1) Randomization process, (2) Deviations from the intended interventions, (3) Missing outcome data, (4) Measurement of the outcome, and (5) Selection of the reported result. Two reviewers (AZ & RS) assessed each study using the previously defined parameters and used the published article, protocols, and trial-registries to inform their judgment of each domain. Responses to signalling questions for each domain are used by the RoB2 algorithm to determine a proposed judgment on the risk of bias for each study. Additionally, the Jadad Scale for Reporting Randomized Controlled Trials and GRADE approach was employed to further interpret the quality of available evidence.^23^^,^^24^ We created funnel plots for our primary outcome measure of change in depression severity to assess publication bias.^25^

### Role of the funding source

The funder of the study had no role in study design, data collection, data analysis, data interpretation, or writing of the report.

## Results

**Fig. 1:**
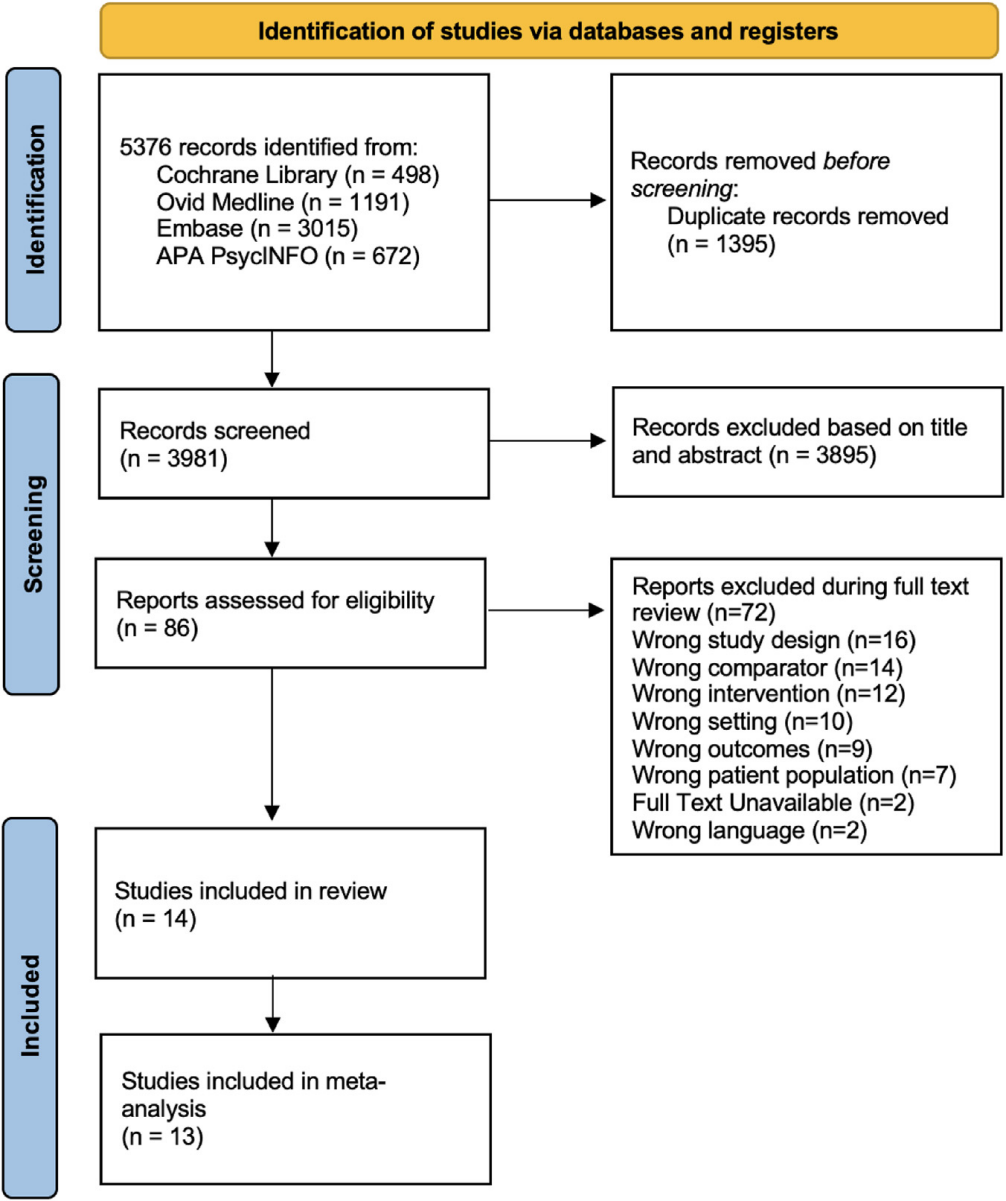
Prisma flow diagram.

The systematic literature review identified a total of 5376 records. This included 498 studies identified through the Cochrane Library, 1191 from MEDLINE, 3015 from Embase, and 672 from PsycINFO. 1395 duplicates were removed before screening, resulting in 3422 unique records screened. 3355 articles were excluded during the title and abstract screening stage, and an additional 54 articles were excluded during full-text screening, resulting in 14 studies included in the systematic review and 13 included studies for SSRI and SNRI meta-analysis, totaling 968 participants with trial sample sizes ranging from n = 22 to 127 (Fig. 1). Table 1 summarizes the baseline sample characteristics of all studies with any medication class included in the review, while Table 2 summarizes baseline sample characteristics of the SSRI and SNRI-treated groups included in the meta-analysis. The HAMD and MADRS were the most commonly used clinical scales to assess efficacy across studies. Most participants in SSRI trials were female (63%), and the SNRI group showed a similar distribution (65.9% female). Included trials tended to enroll midlife adults. The majority (13/14) of studies targeted the left dorsolateral prefrontal cortex with excitatory rTMS protocols with a figure of eight coil with heuristic or EEG based targeting methods, including theta-burst (1 study) and high-frequency stimulation (12 studies), while one study targeted the right dorsolateral prefrontal cortex. Most included studies involved once-daily rTMS and medication treatments, ranging from 10 to 40 sessions. No studies used accelerated protocols, neuronavigation or dTMS. Treatment duration ranged from 2 to 10 weeks, with an average [SD] treatment period of 4 [2] weeks (Table 1).^10^^,^^11^^,^^13^^,^^26^, ^27^, ^28^, ^29^, ^30^, ^31^, ^32^, ^33^, ^34^, ^35^, ^36^

**Table 1:** Summary of study characteristics.

Study + country	Study design	Demographics sample size, mean age (SD), Sex (%F)	Primary diagnosis	Medication/treatment arms	TMS procedure	Clinical outcomes
Chen et al., 2022 China	RCT	N = 100 adolescents (12–18 yrs) rTMS: N = 48; 15 (2.3); 85% F Sham: N = 49; 15 (2.4); 78% F	MDD	1. Sertraline (SSRI) 50 mg + rTMS 2. Sertraline + sham rTMS 3. Increase to 100 mg if no response	10 Hz left DLPFC 10 sessions over 2 weeks Device: Magstim Rapid stimulator with a figure- 8 coil Locating method: 5-cm rule	**ΔHAMD-17** - Active: −11 - Sham: −6.3 **Remission** - Active: 18/49 (37%) - Sham: 6/48 (13%) **Response** - Active: 31/49 (63%) - Sham: 14/48 (29%)
Wang et al., 2017 China	RCT	N = 43; 29 (8.9) rTMS: N = 22; 29 (8.5); 45.5% F Sham: N = 21; 30 (9.5); 52% F	MDD	1. Paroxetine (SSRI) + rTMS 2. Paroxetine + sham TMS Paroxetine dosage: 10 or 20 mg for 1st week, 20 or 30 mg from day 8 on, some took 40 mg from day 29	10 Hz Left DLPFC 20 sessions over 4 weeks Device: Magstim Rapid stimulator with a 70 mm figure- 8 coil Locating method: 5-cm rule	**ΔHAMD-24** - Active: −36 - Sham: −35 **Remission** - Active: 19/22 (86%) - Sham: 16/21 (76%) **Response** - Active: 20/22 (91%) - Sham: 18/21 (86%)
Dai et al., 2020 China	RCT Double-blind	N = 124; 69 (8.7) rTMS: N = 62, 69 (8.7); 63% F Sham: N = 62, 67 (9.9); 60% F	MDD	1. Escitalopram (SSRI) 5 mg/d - 10 mg/d + rTMS 2. 5 mg/d - 10 mg/d Escitalopram + sham rTMS	10 Hz, left DLPFC 20 sessions over 4 weeks Device: Magstim Rapid stimulator with a figure- 8 coil Locating method: n/a	**ΔHAMD-24** - Active: −20 - Sham: −16 **Remission** - Active: n/a - Sham: n/a **Response** - Active: n/a - Sham: n/a
Guan et al., 2021 China	RCT	N = 51 iTBS: N = 27; 31 (7.7); 59% F Sham: N = 24; 29 (7.1); 58% F	MDD	1. Escitalopram (SSRI) 10 mg–20 mg/d + rTMS 2. Escitalopram + sham rTMS	iTBS Oz point of the occipital region 40 sessions over 20 days Device: MagPro R30 stimulator with a 100 mm figure- 8 coil Locating method: 10–20 EEG system	**ΔHAMD** - Active: −9.4 - Sham: −7.5 **Remission** - Active: n/a - Sham: n/a **Response** - Active: n/a - Sham: n/a
García-Toro et al., 2001 Spain	RCT	N = 22 rTMS: N = 11; 43 (13); 54.5% F Sham: N = 11; 45 (18); 54.5% F	MDD	1. Sertraline (SSRI) 50 mg/d for 2 weeks + rTMS 2. Sertraline + sham rTMS	20 Hz left DLPFC 10 sessions over 2 weeks Device: Dantec Magpro stimulator with an 85 mm figure- 8 coil Locating method: 5-cm rule	**ΔHAMD-21** - Active: −12 - Sham: −12 **Remission:** - Active: n/a - Sham: n/a **Response:** - Active: n/a - Sham: n/a
Ahmadpanah et al., 2023 Iran	RCT	N = 40 rTMS: N = 18; 32 (6.8); 60% F Sham: N = 17; 32 (6.9); 55% F	MDD	1. Sertraline (SSRI) 25–100 mg/d for 4 weeks + rTMS 2. Sertraline + sham rTMS	10 Hz left DLPFC 10 sessions Device: 70 mm D70 Air Film Coil from Magstim Locating method: 5-cm rule	**ΔMADRS** - Active: −5.6 - Sham: −3.1 **Remission** - Active: n/a - Sham: n/a **Response** - Active: 15/18 (83%) - Sham: 8/17 (47%)
Bretlau et al., 2008 Denmark	RCT	N = 45 rTMS: N = 22; 53 (10); 68% F Sham: N = 23; 58 (10); 56.5% F	MDD	1. Escitalopram (SSRI) 20 mg/d for 12 weeks + rTMS 2. Escitalopram + sham rTMS	8 Hz left DLPFC 15 sessions over 3 weeks Device: Magstim Super Rapid Stimulator with a 70 mm figure- 8 coil Locating method: 5-cm rule	**ΔHAMD-17** - Active: −14 - Sham: −11 **Remission** - Active: n/a - Sham: n/a **Response** - Active: n/a - Sham: n/a
Huang et al., 2012 China	RCT	N = 56 rTMS: N = 28; 33 (7.28); 61% F Sham: N = 28; 31 (7.39); 71% F	MDD	1. Citalopram (SSRI) 20–40 mg/d for 4 weeks + rTMS 2. Citalopram + sham lf-rTMS	10 Hz left DLPFC 10 sessions over 2 weeks Device: Magstim Rapid stimulator with a figure- 8 coil Locating method: 5-cm rule	**ΔHAMD-17** - Active: −10 - Sham: −7.6 **Remission** - Active: 11/28 (39%) - Sham: 8/28 (29%) **Response** - Active: 13/28 (46%) - Sham: 10/28 (36%)
Wang et al., 2023 China	RCT	N = 120 rTMS: N = 60; 66.6 (8.0); 58.3% F Sham: N = 60; 65.7 (7.2); 68.3% F	MDD	1. Escitalopram (SSRI) 5–20 mg/d for 8 weeks + rTMS 2. Escitalopram (SSRI) + sham rTMS	10 Hz left DLPFC 40 sessions over 8 weeks Device: MagPro magnetic stimulator with a figure- 8 coil Locating method: n/a	**ΔHAMD-17** - Active: - Sham: **Remission** - Active: 23/60 (38.3%) - Sham: 7/60 (11.7%) **Response** - Active: 57/60 (80%) - Sham: 48/60 (95%)
Rossini et al., 2005 Italy	RCT	N = 99; 47 (13); 80% F Venlafaxine + rTMS: N = 17; 49 (16); 82% F Sertraline + rTMS: N = 16; 49 (14); 87.5% F Escitalopram + rTMS: N = 17; 48 (12); 65% F Venlafaxine + sham: N = 17; 42 (11.5); 87.5% F Sertraline + sham: N = 16; 48 (10); 81% F Escitalopram + sham: N = 16; 49 (14); 76.5%	MDD	1. Venlafaxine (SNRI) 225 mg/d + rTMS 2. Sertraline (SSRI) 150 mg/d + rTMS 3. Escitalopram (SSRI) 15 mg/d + rTMS 4. Venlafaxine (SNRI) + sham 5. Sertraline (SSRI) + sham 6. Escitalopram (SSRI) + sham	15 Hz left DLPFC 10 sessions over 2 weeks Device: Magstim Rapid stimulator with a 70 mm figure- 8 coil Locating method: 5-cm rule	**ΔHAMD-17** - Active: n/a - Sham: n/a **Remission (SSRI)** - Active: 21/30 (70%) - Sham: 17/30 (57%) **Remission (SNRI)** - Active: 12/15 (80%) - Sham: 7/14 (50%) **Response (SSRI)** - Active: 22/30 (73%) - Sham: 22/30 (73%) **Response (SNRI)** - Active: 14/15 (93%) - Sham: 10/14 (71%)
Ullrich et al., 2012 Germany	RCT Double Blind	N = 43 UHF rTMS: N = 22; 57 (10); 68% F LF sham rTMS: N = 21; 54 (7.8); 57% F	MDD	1. Venlafaxine (SNRI) + active UHF rTMS 2. Venlafaxine + sham (lf- right DLPFC rTMS)	30 Hz UHF Left DLPFC 15 sessions over 3 weeks Device: Dantec MagPro stimulator with a 100 mm figure- 8 coil Locating method: 10–20 EEG system	**ΔHAMD-21** - Active: −9.3 - Sham: −3.9 **Remission** - Active: 0/22 (0%) - Sham: 0/21 (0%) **Response** - Active: 4/22 (18%) - Sham: 0/21 (0%)
Brunelin et al., 2014 France	RCT	N = 101 rTMS: N = 50; 54 (12); 68% F Sham: N = 51; 56 (9.9); 69% F Placebo: N = 54; Age 53 (11); 63% F	MDD	1. Venlafaxine (SNRI) 150–225 mg/d for 4 weeks + active rTMS 2. Venlafaxine + sham (lf- right DLPFC rTMS) Placebo + active rTMS	1 Hz right DLPFC over 2–6 weeks Device: Magstim Super Rapid stimulator with a 70 mm figure- 8 coil Locating method: 6-cm rule	**ΔHAMD-17** - Active: −11 - Sham: −11 **Remission** - Active: 14/50 (28%) - Sham: 22/51 (43%) - Placebo: 22/54 (41%) **Response** - Active: n/a - Sham: n/a - Placebo: n/a
Herwig et al., 2018 Germany and Austria	RCT Double blind	N = 127 rTMS: N = 62; 50 (15); 71% F Sham: N = 65; 49 (13); 49% F	MDD	1. Venlafaxine (SNRI) 75 mg/d) + active rTMS 2. Venlafaxine + sham rTMS	10 Hz Left DLPFC 15 sessions over 3 weeks Device: Magstim Rapid, Medtronic Magpro, and Medtronic Maglite r25 stimulator with a 70 mm figure- 8 coil Locating method: 10–20 EEG system	**ΔHAMD-21** - Active: −15.5 - Sham: −18 **Remission** - Active: 6/62 (10%) - Sham: 10/65 (15%) **Response** - Active: n/a - Sham: n/a
Pu et al., 2022 China	RCT	N = 100; 35 (5.1) rTMS group: N = 50; 35 (5.1); 62% F Sham group: N = 50; 34 (4.7); 56% F	MDD	1. Agomelatine (melatonin agonist-atypical antidepressant) + hf-rTMS 2. Agomelatine (melatonin agonist-atypical antidepressant) + sham rTMS	High frequency, left DLPFC 20 sessions over 8 weeks Device: coil from Wuhan Iruide Medical Equipment Locating method: n/a	**ΔHAMD-17:** −1.6 - Active: −18 - Sham: −15 **Remission** - Active: n/a - Sham: n/a **Response** - Active: 33/42 (79%) - Sham: 21/40 (53%)

Legend: SD = standard deviation; %F = % female; HAMD = Hamilton Rating Scale Score in Depression; MADRS = Montgomery–Åsberg Depression Rating Scale; DLPFC = dorsolateral prefrontal cortex; RCT = randomized control trial; MDD = Major Depressive Disorder; hf-rTMS = high frequency repetitive transcranial magnetic stimulation; lf-rTMS = low frequency repetitive transcranial magnetic stimulation; UHF = ultra-high frequency; iTBS = intermittent theta burst stimulation; Oz = occipital zero electrode position; n/a = not assessed.

**Table 2:** Baseline study characteristics.

	SSRI Study Characteristics	SNRI Study Characteristics
Studies (total n)	10 Studies (n = 663)	4 Studies (n = 305)
Average sample size/study (±SD)	60 ± 31.5	59 ± 39
Range (participants/study)	22–124	34–127
Age (years)	41 ± 19	52 ± 3.8
% Female	63.5%	66%
Depression scales	HAMD-17 = 6 studies HAMD-21 = 1 study HAMD-24 = 2 studies MADRS = 1 study	HAMD-17 = 2 studies HAMD-21 = 2 study
Baseline HAMD depression score	27 ± 7.5	27 ± 1.7
Baseline MADRS depression score	29 ± 2.8	N/A
rTMS procedure	High-frequency = 8 studies iTBS = 1 study	High-frequency = 3 studies Low-frequency = 1 study
Average number of rTMS treatments in acute series (SD)	16 ± 8.8	16 ± 3.1
Antidepressant type (no. of participants)	Escitalopram (253) Sertraline (194) Citalopram (56) Paroxetine (43)	Venlafaxine (305)

The inclusion criteria across studies demonstrated similar depression severity cut-offs between most SSRI and SNRI studies. Across studies, there was variability in reporting on past treatment history; however, both SSRI and SNRI samples appeared to be evenly matched for treatment-resistance as indexed by the number of prior depressive episodes, duration of episode or illness, and past antidepressant treatment trials (Supplementary Table S1). This suggests that patients included in both SSRI and SNRI samples do not vary with respect to depression severity or resistance to treatment.

Included studies followed a randomized clinical trial design with an rTMS sham-control in MDD. One study consisted of participants who had a comorbid diagnosis of Multiple Sclerosis, one focused on elderly patients, one on adolescents experiencing their first episode of MDD. 10 trials (n = 663) used selective serotonin reuptake inhibitors (SSRI), 4 studies (n = 305) treated participants with serotonin and norepinephrine reuptake inhibitors (SNRI), and 1 study used agomelatine (n = 100). Only one study^33^ had a direct comparison of SSRIs and SNRIs to sham rTMS treatment, whereas all other studies looked at SSRIs and SNRIs, independently (Table 1). The included SSRI studies used escitalopram (n = 253; dose:10–20 mg/d), sertraline (n = 194; dose:25–150 mg/d), citalopram (n = 56; dose:20–40 mg/d), and paroxetine (n = 43; dose:20/40 mg/d), while the SNRI studies all used venlafaxine (n = 305; dose:75–225 mg/d), with prescribed doses varying within studies as well as between studies.

The overall risk of bias for the included studies ranged from low to high. 5 of the 14 studies had some concerns; 1 had a high risk of bias, and the remaining studies had a low risk of bias. The high-risk study did not blind the intervention treatment from assessors and we were unable to locate a pre-specified analysis plan to determine whether there were any deviations.^33^ Of the remaining 5 studies there were some concerns due to deviations from the intended interventions, measurement of outcome data, and the selection of the reported result.^13^^,^^25^^,^^30^^,^^34^^,^^35^ There were no concerns arising from the randomization process or missing outcome data (Supplementary Fig. S1). There was no appreciable bias in measuring primary and secondary outcome data, nor were there any apparent reporting biases when evaluating the study pre-registration reports, protocols, and/or methods/designs papers. The Jadad scale demonstrated good to excellent methodological quality across studies with scores ranging from 4 to 5 (Supplementary Table S2). Additionally, funnel plots were created for both the SSRI and SNRI groups (Supplementary Fig. S2).

**Fig. 2:**
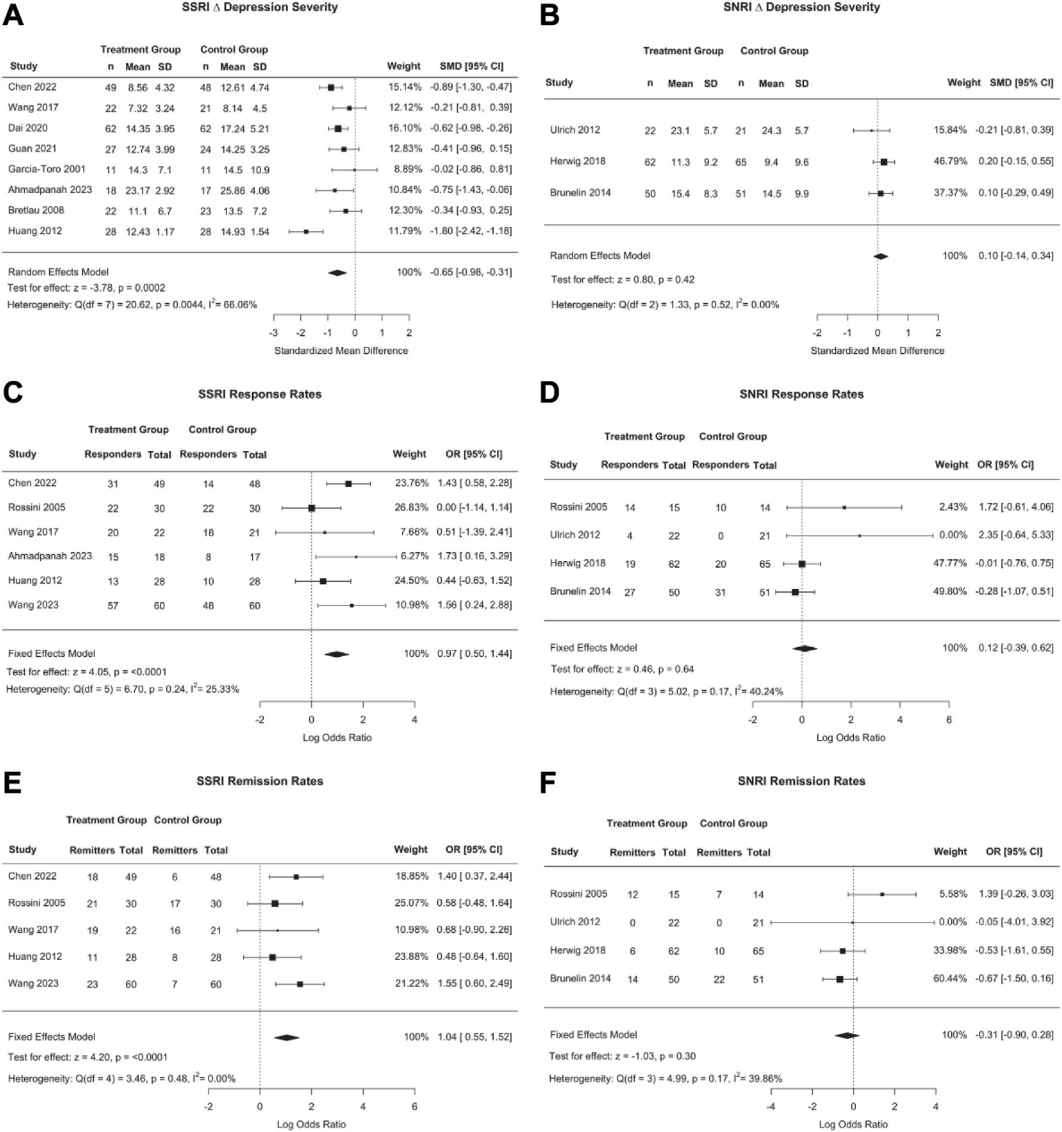
Clinical outcomes between treatment (rTMS + SSRI/SNRI) and control groups (Sham rTMS + SSRI/SNRI). Panels A–F show forest plot for both SSRI (on the left) and SNRI groups (on the right). The first row of forest plots, Panel A and B, shows Standardized Mean Difference (SMD) of depression severity in treatment versus control groups. Panels C, D and E, F show treatment response and remission rates respectively as an OR. Horizontal bars show 95% confidence intervals, with studies closer to the dashed vertical line having no effect on depression severity. Square sizes reflect the weight of the overall study. The Q and I2 statistics indicate heterogeneity, with lower values indicating less heterogeneity. p-values for each measure are also reported. A negative SMD indicated that the treatment group had a greater decline in depression severity than the control group. A positive OR indicated that the treatment group had more patients who responded or remitted to treatment than the control group.

Raw data for all meta-analyses are reported in Supplementary Tables S4–S6. Fig. 2a and b shows forest plots by antidepressant classes, comparing active to sham-based rTMS effects when commenced with antidepressants. There was a significant effect of rTMS and SSRIs (SMD [CI] of −0.65 [−0.98, −0.31], p = 0.0002, Q = 20.62, I^2^ = 66.1%)) on change in depression severity, such that a greater reduction in HAMD and MADRS scores were observed in the active rTMS + SSRI treatment group compared to the control group (drug + sham rTMS). Heterogeneity was high (I^2^ > 60%). There was no effect of combined rTMS and SNRIs on depression severity compared to the sham rTMS group (SMD of 0.10 [−0.14, 0.34], p = 0.42, Q = 1.33, I^2^ = 0.00%).

Subgroup analyses revealed that escitalopram (−0.79 [−1.35, 0.20], p = 0.008, I^2^ = 79.72%) and sertraline (−0.65 [−1.11, −0.19], p = 0.42, I^2^ = 29.49%) significantly decreased depression severity (Supplementary Fig. S3). Meta-regression analyses revealed that neither medication dose (p = 0.81) nor number of rTMS treatment sessions (p = 0.32) were significantly associated with change in depression severity (Supplementary Figs. S4 and S5). Similar to the pooled SSRI analysis, subgroup analyses grouping SSRI + rTMS trials by treatment duration found a significant effect for both studies of ≤4 weeks (−0.79 [−1.33, −0.25], p = 0.004, I^2^ = 74.71%) and >4 weeks in duration (−0.49 [−0.76, −0.22], p = 0.0004, I^2^ = 0.00%) (Supplementary Fig. S6).

One SSRI study assessed patients with first-episode depression.^10^ Sensitivity analyses were performed by excluding this study, and the primary outcome measure revealed similar results to the principal analysis (SMD [CI] of −0.60 [−1.0, −0.21], p = 0.0026, I^2^ = 68.91%) (Supplementary Fig. S7). To further evaluate the effects of outliers on effect size in SSRI studies, we created a radial plot of the primary outcome, change in depression severity (Supplementary Fig. S8). One SSRI study^26^ appeared to be contributing to heterogeneity more substantially. We conducted an additional sensitivity analysis by recomputing the SSRI + rTMS meta-analysis, and reassessing heterogeneity with this study removed (Supplementary Fig. S9). A similar result was found to that of the main analysis (SMD [CI] of −0.55 [−0.75, −0.34], p < 0.0001), and heterogeneity was low and non-significant among the remaining SSRI studies (Q = 6.52, I^2^ = 7.98%, p = 0.37).

Fig. 2c and d shows forest plots of ORs for treatment response. The model demonstrated significantly greater response rates following treatment with rTMS and SSRI versus sham rTMS and SSRI (OR = 0.97 [0.50, 1.44], p < 0.0001, Q = 6.7, I^2^ = 25.33%). There was no effect of combined rTMS and SNRIs on response rates (OR = 0.12 [−0.39, 0.62], p = 0.64, I^2^ = 0.00%). When the sample was restricted to treatment-resistant samples, by excluding an SSRI study of first-episode depression, sensitivity analyses showed a similar significant result with lower heterogeneity (SMD [CI] of 0.77 [−0.09, 1.20], p = 0.0075, I^2^ = 19.56%) (Supplementary Fig. S7).

Fig. 2e and f shows forest plots of ORs for treatment remission, a dichotomous variable. The model demonstrated significantly greater remission rates following active rTMS and SSRI versus sham rTMS and SSRI treatment (OR = 1.04 [0.55, 1.52], p < 0.0001, Q = 3.46, I^2^ = 0.00%). There was no effect of combined rTMS and SNRIs on remission rates (p = 0.86) with an (OR = −0.31 [−0.90, 0.28], Q = 5.0, I^2^ = 39.9%). Results remained similar when restricting the analysis to treatment-resistant samples (Supplementary Fig. S7).

**Fig. 3:**
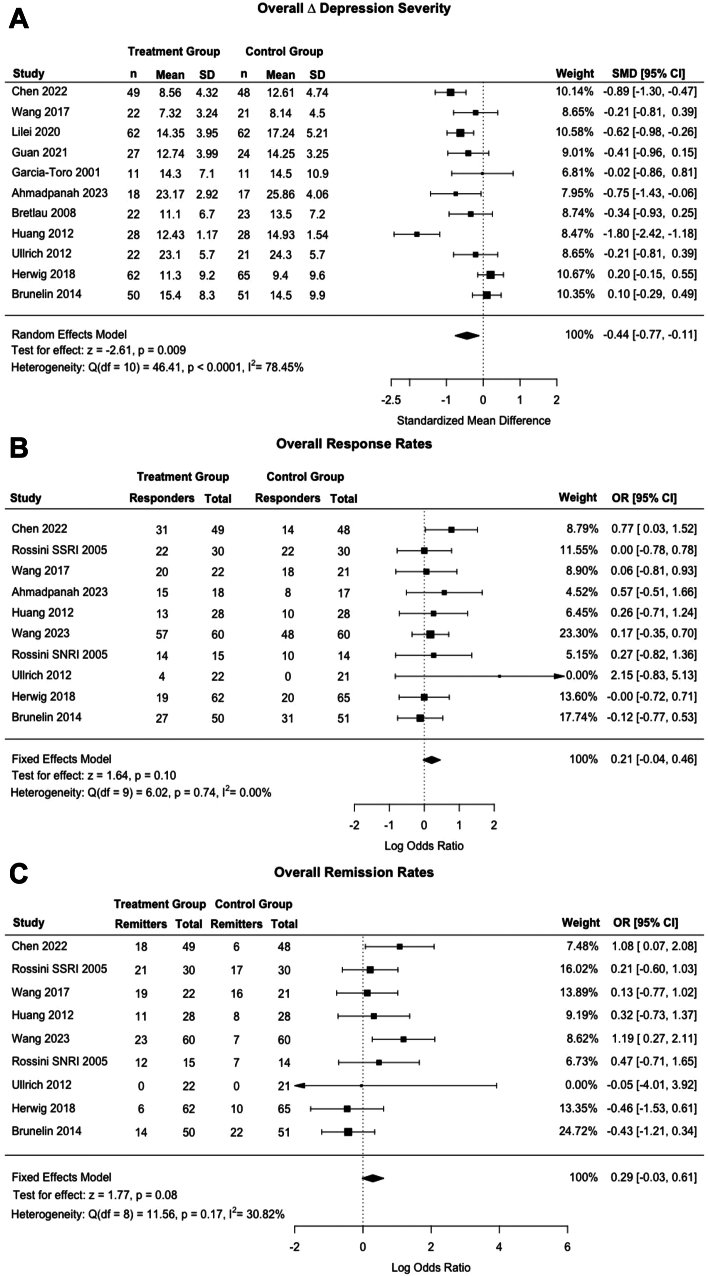
Clinical outcomes in treatment (rTMS + Adjunctive Medication) and control groups across all medication classes (Sham rTMS + Adjunctive Medication) Panel A shows a forest plot of Standardized Mean Difference (SMD) of depression severity in treatment versus control groups. The second forest plot (B) shows response rates of treatment and control groups. Finally, panel C shows a forest plot of remission rates of treatment and control. Horizontal bars show 95% confidence intervals, with studies closer to the dashed vertical line having no effect on depression severity. Square sizes reflect the weight of the overall study. The Q and I2 statistics indicate heterogeneity, with lower values indicating less heterogeneity. p-values for each measure are also reported. A negative SMD indicated that the treatment group had a greater decline in depression severity than the control group. A positive OR indicated that the treatment group had a greater number of patients who responded or remitted following treatment than the control group.

We performed a secondary meta-analysis of the effect of all antidepressants on clinical outcomes, in which all sham-controlled studies were combined prospectively with both SSRI and SNRI medication. For this meta-analysis, a total of 13 studies were included. An additional paper assessing the efficacy of agomelatine (melatonin agonist) was included in the systematic review but not the meta-analysis.^34^ Fig. 3a shows a forest plot of SMDs of follow-up depression severity scores of the treatment group (active rTMS + medication) versus the control group (sham rTMS + medication), pooling across all studies. There was a significant negative effect of combined rTMS and antidepressant medication (p = 0.009) on change in depression severity, which represents a greater reduction in HAMD and MADRS scores compared to control with an SMD [CI] of −0.44 [−0.77, −0.11]. Heterogeneity was high (Q = 46.4, I^2^ = 78%).

Fig. 3b shows a forest plot of ORs for treatment response, a dichotomous variable. The model demonstrated no effect of active rTMS and antidepressant medication versus sham TMS and antidepressant medication treatment on response rates (OR = 0.21 [−0.04, 0.46], p = 0.10). Heterogeneity was low (Q = 6.02, I^2^ = 0.00%).

Fig. 3c shows a forest plot of ORs for treatment remission, a dichotomous variable. The model demonstrated a non-significant increase in remission rates following active rTMS and medication versus sham rTMS and medication treatment ((OR = 0.29 [−0.03, 0.61], p = 0.08). Heterogeneity was low (Q = 11.56, I^2^ = 30.82%).

Subgroup analyses revealed that there was a significant effect for combined antidepressants and active rTMS versus sham rTMS on change in depression severity scores across all studies with ≤4 weeks of treatment (−0.54 [−1.06, −0.02], p = 0.042, I^2^ = 84.33%), and no significant differences were found for other outcomes/treatment durations (Supplementary Fig. S10).

The number of adverse events was divided by the total number of patients in each group. There were no incidents of seizures or manic episodes across studies. Only one (SNRI) study reported that four patients had a psychiatric admission during treatment (3 active and 1 sham) (Supplementary Table S6).^27^ There appears to be no difference in the number of other adverse events between active rTMS + medication and sham rTMS + medication groups.

## Discussion

To our knowledge, this was the first study to evaluate by meta-analysis the clinical effects of antidepressant class when initiated concurrently with rTMS in prospective, randomized, sham-controlled trials in MDD. In a sample size of 968 individuals, we found a medium to large effect for antidepressant efficacy in our primary outcome measure of depression severity when rTMS was prospectively combined with SSRIs compared to SSRI monotherapy (i.e., with sham rTMS). No significant differences were observed when rTMS was combined with SNRIs compared to initiating an SNRI alone. Further, when antidepressants were pooled into a single class, we found an effect for change in antidepressant score but not response and remission rates compared to medication alone. There were no differences with respect to side effects between SSRI and SNRI groups. Caution is required when interpreting these results, as we found high heterogeneity in some analyses (SSRI trials and pooling across all antidepressants). Sensitivity analysis revealed that a single study with a high effect size contributed to higher heterogeneity measures. Removal of this study did not alter the main findings and substantially reduced the heterogeneity. Although inclusion criteria varied across studies, there was overall evidence that both SNRI and SSRI trials contained individuals with treatment-resistant depression of at least moderate severity that was not responsive to prior antidepressant treatments. Only one study examined combination therapy in first-episode MDD, and removing this study from the analysis did not alter the primary findings. Since both groups had a similar sex distribution, we did not perform additional subgroup analyses analyzing the effect of sex. Additionally, the meta-regression and subgroup analyses demonstrated additional stimulations, duration of treatment (≤/>4 weeks), and higher medication doses did not affect the main findings. These data suggest that rTMS provides enhanced antidepressant effects for SSRIs when commenced in combination and that combination therapy is not equivalent across antidepressant classes. This has significant clinical implications and merits consideration for future rTMS clinical trial design.

While early rTMS clinical trials evaluated rTMS efficacy as a monotherapy, they did not permit an assessment of combination therapy with antidepressants. The majority of recent rTMS trials enrolled patients on stable doses of antidepressants and did not systematically initiate medication with rTMS. An earlier meta-analysis suggested an enhanced treatment effect when combining rTMS with antidepressants.^34^ These findings were confirmed by two meta-analyses of prospective randomized trials, combining antidepressants and rTMS. These studies demonstrated a therapeutic advantage when antidepressants were combined with rTMS compared to medication alone.^37^^,^^38^ The current meta-analysis considered prospective randomized trials that commenced a specified antidepressant with rTMS and demonstrated an additive benefit of acute combination therapy on reducing depression symptoms but no significant effects on remission and response rates compared to initiating medication alone. Moreover, we extend previous meta-analyses and demonstrate that SSRIs enhance clinical outcomes with adjunctive rTMS, while SNRIs had no additive clinical benefits to taking the drug alone.

These findings are clinically important and call into question previous efficacy results from clinical trials that did not control for specific antidepressant medications. Future rTMS trials should consider the type of antidepressant used during the trial. Different antidepressant classes may possibly impact the efficacy of other neuromodulation and brain stimulation treatments, a topic which has not been well studied.^38^ Treatment resistant patients who previously did not respond to rTMS treatment may benefit from initiating a low dose SSRI in combination with an acute rTMS protocol with fewer stimulation sessions, which could also increase tolerability to treatment.

The mechanistic underpinning of this synergistic effect may relate to greater neuroplastic effects of SSRIs than SNRIs. Several neurophysiological studies have found that rTMS when used concurrently with SSRIs, increases cortical excitability; however, SNRIs have not been associated with neuroplasticity or cortical excitability.^39^, ^40^, ^41^, ^42^, ^43^ Although both drugs work at the presynaptic membrane level and inhibit the reuptake of biogenic amines,^44^^,^^45^ the effect of SSRIs on intracortical inhibition may be associated with the initial, partial GABAergic effect of the drug.^46^ Similar effects may be observed in SNRIs such as venlafaxine, which has demonstrated selective 5-HT reuptake inhibitor action at low doses and dual serotonin and norepinephrine reuptake inhibition at high doses.^47^

Our study was limited to summary statistics and did not permit an evaluation of combination therapy effects at the individual patient level. Our findings are only generalizable to the acute treatment phase and did not consider continuation therapy. Insufficient data was available to report on long-term treatment outcomes, maintenance therapy, or to conduct a network meta-analysis. Although several studies retrospectively assessed rTMS and concurrent medication use with various psychotropic medication classes (i.e., TCAs, MAOIs, bupropion, mirtazapine, ketamine, antipsychotics, stimulants, and benzodiazepines), there were insufficient prospective randomized control studies that examined these other classes of antidepressants with acute rTMS. Similarly, there were fewer studies assessing the effects of SNRIs and the included studies only assessed the effect of venlafaxine. Retrospective, open-label and/or naturalistic studies were not included. Further, we could not assess whether rTMS combined with medication was more effective than rTMS alone due to a lack of trials with a placebo-medication controlled arm.

There was heterogeneity between studies when reporting measures that index treatment-resistance; however, the majority of SSRI and SNRI trials reported either long duration of illness, multiple past antidepressant trials, and/or a history of recurrent depressive episodes. There was also a wide age range due to the inclusion of studies that assessed both adolescents and patients with late-life depression. Previous studies have demonstrated that elderly patients show a reduced response to conventional rTMS treatment compared to younger adults.^48^ It is possible that the SSRI group, which had a lower average age of study participants, may have benefitted from this effect. However, we ran a sensitivity analysis that did not reveal changes in the primary outcome (change in formal depression scale scores) when removing a study in first-episode adolescent depression. Previous retrospective analyses in older adults that combined an SSRI with rTMS in post-stroke depression found a similar synergistic effect when combining rTMS + SSRIs compared to either escitalopram,^16^ sertraline,^15^ and paroxetine alone.^14^ However, when excluding the adolescent first-episode depression sample, only a trend was found for response and remission rate outcome measures. rTMS and medication regimens in the included studies varied in protocol (number of rTMS treatments, duration of treatment, and stimulation parameters), all of which may have affected clinical outcomes. However, all studies except one included high-frequency left DLPFC rTMS and the average number of treatments across trials was 16 for both the SNRI and SSRI study groups. Moreover, for the SSRI group, we found similar effects for combination therapy in studies delivering treatment for ≤4 weeks and >4 weeks in duration.

Large, randomized, and placebo/sham-controlled trials are warranted to explore whether combination antidepressant and rTMS therapy differs based on stimulation protocol, degree of treatment resistance, and/or comorbid psychiatric disorders. Longer-term, prospective treatment trials examining continuation therapy among different antidepressant classes would also help to inform antidepressant treatment choice. Neurophysiological studies using paired/single-pulse TMS paradigms and functional brain imaging are required to understand the neuronal substrates underlying SSRI and rTMS therapy in MDD.

In summary, we found a large significant decline in formal depression scores when rTMS is combined with SSRIs compared to SSRI monotherapy and SNRIs in combination with rTMS. While clinical guidelines comment on the use of medications that can negatively affect treatment response,^49^ our findings support the use of SSRIs as a class when initiating a medication change at the onset of rTMS therapy.

## Contributors

AZ, RS, PG, JSR, MG, CH, WS, BD, NL, and SMN assisted with study conceptualization. AZ, RS, and IJS assisted with the literature search. AZ, RS, and SMN were involved in data extraction and verification. IJS assisted with the code for statistical analysis. AZ assisted with the statistical analysis/meta-analysis. XC Reviewed the results and appropriate statistical methods used for meta-analyses. AZ created figures. AZ, RS, IJS, PG, JR, MG, XC, CH, WS, BJD, NL, and SNM contributed to the first draft of the manuscript. All authors assisted in editing the manuscript and have read and approved the final version of the manuscript. SMN oversaw the project, supervision of the research team, and acted as a tie breaker for article inclusion. The corresponding author had full access to all the data in the study and had final responsibility for the decision to submit for publication.

## Data sharing statement

As this meta-analysis consisted of already published data, individual participant data is not available. Raw data used for the meta-analysis is included in the supplement. Software and packages used to perform all statistical analyses are publicly available (R statistical software and packages included tidyverse, meta, metafor, and ggplot2).

## Declaration of interests

SMN has received research support from Brain Canada, CIHR, the Brain and Behaviour Research Foundation, Tory Trauma Program at Sunnybrook Health Sciences Centre, and he is affiliated with the Ontario Ketamine and Infusion Clinic. He reports no conflicts of interest with the current work.

PG has received grants in the area from CIHR and Physician Services Incorporated. He is the DSMB chair for an intravenous ketamine for life depression study. He receives salary support from the University of Toronto, Department of Psychiatry, Academic Scholar Award; he has provided consulting services for Abbott. He reports no conflicts of interest with the current work.

JSR has received support from the Harquail Centre for Neuromodulation, the Dr. Sandra Black Centre for Brain Resilience & Recovery and CIHR. She reports no conflicts of interest with the current work.

CH has received support from the Harquail Centre for Neuromodulation and CIHR. He reports no conflicts of interest with the current work.

WS has received support from CIHR, NSERC, the Alzheimer’s Association & Brain Canada, Weston Brain Institute & Alzheimer’s Research UK, Alzheimer’s Association, and Michael J. Fox Foundation. He reports no conflicts of interest with the current work. All other authors (AZ, RS, IJS, CH, WS, XC, MG, BJD, NL) have no disclosures and report no conflicts of interest.

## Appendix ASupplementary data

Supplementary_Information_Results

## References

[1] Cosmo C., Zandvakili A., Petrosino N.J., Berlow Y.A., Philip N.S. (2021). Repetitive transcranial magnetic stimulation for treatment-resistant depression: recent critical advances in patient care. Curr Treat Options Psych.

[2] Voigt J., Carpenter L., Leuchter A. (2019). A systematic literature review of the clinical efficacy of repetitive transcranial magnetic stimulation (rTMS) in non-treatment resistant patients with major depressive disorder. BMC Psychiatr.

[3] Kishi T., Ikuta T., Sakuma K. (2023). Antidepressants for the treatment of adults with major depressive disorder in the maintenance phase: a systematic review and network meta-analysis. Mol Psychiatry.

[4] Wen K.S., Zheng W. (2024). Optimization strategies of transcranial magnetic stimulation in major depressive disorder. Alpha Psychiatry.

[5] Cipriani A., Furukawa T.A., Salanti G. (2018). Comparative efficacy and acceptability of 21 antidepressant drugs for the acute treatment of adults with major depressive disorder: a systematic review and network meta-analysis. Lancet.

[6] Rachid F. (2018). Maintenance repetitive transcranial magnetic stimulation (rTMS) for relapse prevention in with depression: a review. Psychiatr Res.

[7] Haesebaert F., Moirand R., Schott-Pethelaz A.M., Brunelin J., Poulet E. (2018). Usefulness of repetitive transcranial magnetic stimulation as a maintenance treatment in patients with major depression. World J Biol Psychiatr.

[8] Blumberger D.M., Vila-Rodriguez F., Thorpe K.E. (2018). Effectiveness of theta burst versus high-frequency repetitive transcranial magnetic stimulation in patients with depression (THREE-D): a randomised non-inferiority trial. Lancet.

[9] Milev R.V., Giacobbe P., Kennedy S.H. (2016). Canadian network for mood and anxiety treatments (CANMAT) 2016 clinical guidelines for the management of adults with major depressive disorder: section 4. Neurostimulation treatments. Can J Psychiatry.

[10] Chen H., Hu X., Gao J., Han H., Wang X., Xue C. (2022). Early effects of repetitive transcranial magnetic stimulation combined with sertraline in adolescents with first-episode major depressive disorder. Front Psychiatr.

[11] Dai L., Wang P., Zhang P. (2020). The therapeutic effect of repetitive transcranial magnetic stimulation in elderly depression patients. Medicine.

[12] Zhang T., Zhu J., Xu L. (2019). Add-on rTMS for the acute treatment of depressive symptoms is probably more effective in adolescents than in adults: evidence from real-world clinical practice. Brain Stimul.

[13] Ahmadpanah M., Amini S., Mazdeh M. (2023). Effectiveness of repetitive transcranial magnetic stimulation (rTMS) add-on therapy to a standard treatment in individuals with multiple sclerosis and concomitant symptoms of depression—results from a randomized clinical trial and pilot study. J Clin Med.

[14] Liu W., Ding W. (2020). Study on the efficacy and mechanism of paroxetine hydrochloride combined with repetitive transcranial magnetic stimulation in the treatment of post-stroke depression. Int J Clin Exp Med.

[15] Yu F., He R. (2021). The effect of fluoxetine combined with repetitive transcranial magnetic stimulation on the psychological emotions and cognitive and neurological functions of acute post-stroke depression patients. Am J Transl Res.

[16] Zhu Z., Zhu H.X., Jing S.W. (2022). Effect of transcranial magnetic stimulation in combination with citalopram on patients with post-stroke depression. Front Hum Neurosci.

[17] Page M.J., McKenzie J.E., Bossuyt P.M. (2021). The PRISMA 2020 statement: an updated guideline for reporting systematic reviews. BMJ.

[18] DerSimonian R., Laird N. (1986). Meta-analysis in clinical trials. Contr Clin Trials.

[19] Hayasaka Y., Purgato M., Magni L.R. (2015). Dose equivalents of antidepressants: evidence-based recommendations from randomized controlled trials. J Affect Disord.

[20] Cochran W.G. (1950). The comparison of percentages in matched samples. Biometrika.

[21] Higgins J.P.T., Thompson S.G., Deeks J.J., Altman D.G. (2003). Measuring inconsistency in meta-analyses. BMJ.

[22] Sterne J.A.C., Savović J., Page M.J. (2019). RoB 2: a revised tool for assessing risk of bias in randomised trials. BMJ.

[23] Jadad A.R., Moore R.A., Carroll D. (1996). Assessing the quality of reports of randomized clinical trials: is blinding necessary?. Contr Clin Trials.

[24] Guyatt G.H., Oxman A.D., Vist G.E. (2008). GRADE: an emerging consensus on rating quality of evidence and strength of recommendations. BMJ.

[25] Suurmond R., van Rhee H., Hak T. (2017). Introduction, comparison, and validation of Meta-Essentials: a free and simple tool for meta-analysis. Res Synth Methods.

[26] Huang M li, yan Luo B., bo Hu J. (2012). Repetitive transcranial magnetic stimulation in combination with citalopram in young patients with first-episode major depressive disorder: a double-blind, randomized, sham-controlled trial. Aust N Z J Psychiatry.

[27] Brunelin J., Jalenques I., Trojak B. (2014). The efficacy and safety of low frequency repetitive transcranial magnetic stimulation for treatment-resistant depression: the results from a large multicenter French RCT. Brain Stimul.

[28] Bretlau L.G., Lunde M., Lindberg L., Undén M., Dissing S., Bech P. (2008). Repetitive transcranial magnetic stimulation (rTMS) in combination with escitalopram in patients with treatment-resistant major depression. A double-blind, randomised, sham-controlled trial. Pharmacopsychiatry.

[29] Ullrich H., Kranaster L., Sigges E., Andrich J., Sartorius A. (2012). Ultra-high-frequency left prefrontal transcranial magnetic stimulation as augmentation in severely ill patients with depression: a naturalistic sham-controlled, double-blind, randomized trial. Neuropsychobiology.

[30] García-Toro M., Pascual-Leone A., Romera M. (2001). Prefrontal repetitive transcranial magnetic stimulation as add on treatment in depression. J Neurol Neurosurg Psychiatr.

[31] Guan M., Liu X., Guo L. (2021). Improved pre-attentive processing with occipital rTMS treatment in major depressive disorder patients revealed by MMN. Front Hum Neurosci.

[32] Wang Y.M., Li N., Yang L.L. (2017). Randomized controlled trial of repetitive transcranial magnetic stimulation combined with paroxetine for the treatment of patients with first-episode major depressive disorder. Psychiatr Res.

[33] Rossini D., Magri L., Lucca A., Giordani S., Smeraldi E., Zanardi R. (2005). Does rTMS hasten the response to escitalopram, sertraline, or venlafaxine in patients with major depressive disorder? A double-blind, randomized, sham-controlled trial. J Clin Psychiatry.

[34] Pu Z., Hou Q., Yan H., Lin Y., Guo Z. (2023). Efficacy of repetitive transcranial magnetic stimulation and agomelatine on sleep quality and biomarkers of adult patients with mild to moderate depressive disorder. J Affect Disord.

[35] Herwig U., Fallgatter A.J., Höppner J. (2007). Antidepressant effects of augmentative transcranial magnetic stimulation. Br J Psychiatry.

[36] Wang X., Fan X., Zhang L., Liu X., Ji Z. (2023). Repetitive transcranial magnetic stimulation in the treatment of middle-aged and elderly major depressive disorder: a randomized controlled trial. Medicine.

[37] Wei Y., Zhu J., Pan S., Su H., Li H., Wang J. (2017). Meta-analysis of the efficacy and safety of repetitive transcranial magnetic stimulation (rTMS) in the treatment of depression. Shanghai Arch Psychiatry.

[38] Berlim M.T., den Eynde F.V., Daskalakis Z.J. (2013). High-frequency repetitive transcranial magnetic stimulation accelerates and enhances the clinical response to antidepressants in major depression: a meta-analysis of randomized, double-blind, and sham-controlled trials. J Clin Psychiatry.

[39] Maneeton B., Maneeton N., Woottiluk P., Likhitsathian S. (2020). Repetitive transcranial magnetic stimulation combined with anti-depressants for the first episode of major depressive disorder. Curr Neuropharmacol.

[40] Khedr E.M., Elserogy Y., Fawzy M., Abdelrahman A.A., Galal A.M., Noaman M.M. (2020). Effect of psychotropic drugs on cortical excitability of patients with major depressive disorders: a transcranial magnetic stimulation study. Psychiatr Res.

[41] Batsikadze G., Paulus W., Kuo M.F., Nitsche M.A. (2013). Effect of serotonin on paired associative stimulation-induced plasticity in the human motor cortex. Neuropsychopharmacology.

[42] Gerdelat-Mas A., Loubinoux I., Tombari D., Rascol O., Chollet F., Simonetta-Moreau M. (2005). Chronic administration of selective serotonin reuptake inhibitor (SSRI) paroxetine modulates human motor cortex excitability in healthy subjects. Neuroimage.

[43] Lissemore J.I., Mulsant B.H., Rajji T.K. (2021). Cortical inhibition, facilitation and plasticity in late-life depression: effects of venlafaxine pharmacotherapy. J Psychiatr Neurosci.

[44] Baldessarini R.J. (1989). Current status of antidepressants: clinical pharmacology and therapy. J Clin Psychiatry.

[45] Baldessarini R.J. (1984). Treatment of depression by altering monoamine metabolism: precursors and metabolic inhibitors. Psychopharmacol Bull.

[46] Bezchlibnyk-Butler K., Aleksic I., Kennedy S.H. (2000). Citalopram--a review of pharmacological and clinical effects. J Psychiatry Neurosci.

[47] Debonnel G., Saint-André É., Hébert C., de Montigny C., Lavoie N., Blier P. (2006). Differential physiological effects of a low dose and high doses of venlafaxine in major depression. Int J Neuropsychopharmacol.

[48] Pallanti S., Cantisani A., Grassi G. (2012). rTMS age-dependent response in treatment-resistant depressed subjects: a mini-review. CNS Spectr.

[49] Rossi S., Hallett M., Rossini P.M., Pascual-Leone A. (2009). Safety, ethical considerations, and application guidelines for the use of transcranial magnetic stimulation in clinical practice and research. Clin Neurophysiol.

