# Supplementary material for: Antidepressant class and concurrent rTMS outcomes in major depressive disorder: a systematic review and meta-analysis
Source: eClinicalMedicine. 2024 Jul 27;75:102760. doi: 10.1016/j.eclinm.2024.102760 (PMC11338161; doi:10.1016/j.eclinm.2024.102760)
Supplement: Supplementary_Information_Results [file mmc1.docx]

**Supplementary Results**

**Supplementary Tables:**

**Supplementary Table 1.** Table Summarising Data Related to Treatment Resistance for Studies Included in the Meta-Analysis

**Supplementary Table 2.** Results From the Jadad Scale for Reporting Randomized Controlled Trials

**Supplementary Table 3.** Raw Data for Meta-Analysis of Change in Depression Severity

**Supplementary Table 4.** Raw Data for Meta-Analysis of Response

**Supplementary Table 5.** Raw Data for Meta-Analysis of Remission

**Supplementary Table 6.** Table Summarising Data Related to the Safety and Tolerability of Studies Included in the Meta-Analysis

**Supplementary Figures**

**Supplementary Figure 1:** Study Level Cochrane Risk of Bias Analysis Results**Supplementary Figure 2**: Funnel Plot of Primary Outcome Variable ∆ in Depression Severity

**Supplementary Figure 3.** Subgroup Analysis of Effect of SSRI Type on ∆ in Depression Severity

**Supplementary Figure 4:** Meta-Regression of Number of rTMS Treatment Sessions

**Supplementary Figure 5:** Meta-Regression of Medication Dose

**Supplementary Figure 6:** Subgroup Analysis of Effect of Treatment Duration on Treatment Outcomes in SSRI Studies

**Supplementary Figure 7:** Sensitivity Analysis Showing Updated Results with Chen et al. Study Removed

**Supplementary Figure 8:** Radial Plot of Primary Outcome Variable ∆ in Depression Severity in SSRI Studies

**Supplementary Figure 9:** Sensitivity Analysis of Primary Outcome with Huang et al. Study Removed

**Supplementary Figure 10:** Subgroup Analysis of Effect of Treatment Duration on Treatment Outcomes for All Studies

**Supplementary Table 1.** Data related to treatment resistance in studies included in meta-analysis

| **Study** | **Number of failed treatment attempts (SD)** | **Specific Medications** | **Duration in years (SD)** | **Duration of current episode in months (SD)** | **# of previous depressive episodes (SD)** | **Inclusion criteria (Depression severity, etc.)** |
| --- | --- | --- | --- | --- | --- | --- |
| Chen et al., 2022 | ·· | Sertraline | ·· | Control:16 (17) Active: 18 (14) | ·· | First episode, HAMD-17 score > 17 |
| Wang et al., 2017 | ·· | Paroxetine | ·· | Control: 4·3 (1·8) Active: 4·3 (2.3) | ·· | ·· |
| Dai et al., 2020 | ·· | Escitalopram | ·· | N/R | ·· | HAMD score ≥20 |
| Guan et al., 2021 | ·· | Escitalopram | ·· | Control: 4.4 (3.4) Active: 2·9 (2·1) | ·· | ·· |
| García-Toro et al., 2001 | Control: 6  Active: 5 | Sertraline + 26/28 were taking benzodiazepines | ·· | Control: 8·1 (13) Active: 6·6 (4.2) | ·· | ·· |
| Ahmadpanah et al., 2023 | ·· | Sertraline | Control: 7·7 (2·0)  Active: 8·9 (2·9) | ·· | ·· | ·· |
| Bretlau et al., 2008 | In the current episode mean (sd):  Control: 2·5 (0·9) Active: 2·8 (0·9) | Escitalopram, 16/45 taking oxazepam | Control: 18 (15) Active:  16 (13) | Control: 11 (4·6) Active:  12 (3·3) | pts with: ≥6  Control: 13 Active: 8 | failed to respond to ≥1 previous adequate antidepressant treatments during the current depressive episode |
| Huang et al., 2012 | ·· | Citalopram | ·· | Control: 9·7 (3) Active: 9·8 (2·9) | ·· | HAMD-17 score >18 |
| Wang et al., 2023 | .. | Escitalopram | Control: 4·5(1·6)  Active: 4·7(1·4) | .. | .. | Between 45-79 years old |
| Rossini et al., 2005 (SSRI) | ·· | Escitalopram, sertraline | ·· | ·· | Control:  Escitalopram: 3·8 (2.4)  Sertraline: 3·8 (2.4)  Active:  Escitalopram: 3.2 (2.4)  Sertraline: 2·5 (1.4) | HAMD-17 score ≥21 |
| Rossini et al., 2005 (SNRI) | ·· | Venlafaxine | ·· | ·· | Control: 3·0 (2·8) Active: 3·5 (3·6) | HAMD-17 score ≥21 |
| Ullrich et al., 2012 | ·· | Venlafaxine + lithium, lorazepam, and antipsychotics allowed | Control: 6.4 (6·0) Active: 6·9 (3.4) | ·· | ·· | HAMD-21 score ≥20 |
| Brunelin et al., 2014 | ·· | Venlafaxine | Control: 20·5 (11) Active: 17 (12) | Control: 22 (22) Active: 14 (14) | ·· | HAMD-17 score ≥20, excluded patients who previously received rTMS and did not respond to venlafaxine during the current episode |
| Herwig et al., 2018 | Treatment resistance present  Control: 10/65  Active: 9/62 | Venlafaxine + lithium allowed | ·· | ·· | Control:   - 1-3 previous episodes: 36 - ≥4 previous episodes: 29   Active:   - 1-3 previous episodes: 26 - ≥4 previous episodes: 36 | HAMD-21 score ≥18 |

HAMD=Hamilton Rating Scale Score in Depression

**Supplementary Table 2.** Results from the Jadad Scale for Reporting Randomized Controlled Trials

| **Author** | **Year** | **Randomization mentioned** | **Randomization appropriate** | **Blinding mentioned** | **Blinding appropriate** | **Account of all patients** | **Total points** |
| --- | --- | --- | --- | --- | --- | --- | --- |
| Chen | 2022 | 1 | 1 | 1 | 0 | 1 | 4 |
| Wang | 2017 | 1 | 1 | 1 | 1 | 1 | 5 |
| Dai | 2020 | 1 | 1 | 1 | 1 | 1 | 5 |
| Guan | 2021 | 1 | 1 | 1 | 1 | 0 | 4 |
| Garcia-Toro | 2001 | 1 | 0 | 1 | 1 | 1 | 4 |
| Ahmadpanah | 2023 | 1 | 1 | 1 | 1 | 1 | 5 |
| Bretlau | 2008 | 1 | 0 | 1 | 1 | 1 | 4 |
| Huang | 2012 | 1 | 1 | 1 | 1 | 1 | 5 |
| Wang | 2023 | 1 | 1 | 1 | 0 | 1 | 4 |
| Rossini | 2005 | 1 | 1 | 1 | 1 | 1 | 5 |
| Ullrich | 2012 | 1 | 0 | 1 | 1 | 1 | 4 |
| Brunelin | 2014 | 1 | 0 | 1 | 1 | 1 | 4 |
| Herwig | 2018 | 1 | 1 | 1 | 1 | 1 | 5 |
| Pu | 2022 | 1 | 1 | 1 | 1 | 1 | 5 |

**Supplementary Table 3.** Raw data used in change in depression severity analysis

| **Author** | **Year** | **Scale** | **Active**  **N** | **Active Baseline Score** | **Active Baseline SD** | **Active Endpoint** | **Active Endpoint SD** | **Control N** | **Control Baseline**  **Score** | **Control Baseline SD** | **Control Endpoint**  **Score** | **Control endpoint SD** |
| --- | --- | --- | --- | --- | --- | --- | --- | --- | --- | --- | --- | --- |
| Chen | 2022 | hamd-17 | 49 | 19·1 | 3·42 | 8·56 | 4·32 | 48 | 18·88 | 3·33 | 12·61 | 4·74 |
| Wang | 2017 | hamd-24 | 22 | 43·5 | 9·89 | 7·32 | 3·24 | 21 | 42·81 | 9·29 | 8·14 | 4·5 |
| Dai | 2020 | hamd-24 | 62 | 33·97 | 4·95 | 14·35 | 3·95 | 62 | 33·43 | 5·36 | 17·24 | 5·21 |
| Guan | 2021 | hamd | 27 | 22·11 | 4·1 | 12·74 | 3·99 | 24 | 21·71 | 3·605 | 14·25 | 3·25 |
| Garcia-Toro | 2001 | hamd-21 | 11 | 25·9 | 6·4 | 14·3 | 7·1 | 11 | 26·6 | 6·4 | 14·5 | 10·9 |
| Ahmadpanah | 2023 | MADRS | 18 | 28·72 | 2·72 | 23·17 | 2·92 | 17 | 28·94 | 2·97 | 25·86 | 4·06 |
| Bretlau | 2008 | hamd-17 | 22 | 25·3 | 3·0 | 11·1 | 6·7 | 23 | 24·7 | 3·2 | 13·5 | 7·2 |
| Huang | 2012 | hamd-17 | 28 | 22·71 | 2·27 | 12·43 | 1·17 | 28 | 22·5 | 2·71 | 14·93 | 1·54 |
| Wang | 2023 | hamd-17 | 60 | 29·73 | 2·39 | 13·00 |  | 60 | 29·47 | 2·46 | 17·00 |  |
| Ullrich | 2012 | hamd-21 | 22 | 32·4 | 4·8 | 23·1 | 5·7 | 21 | 28·2 | 3·9 | 24·3 | 5·7 |
| Herwig | 2018 | hamd-21 | 62 | 26·8 | 8·9 | 11·3 | 9·2 | 65 | 27 | 10·3 | 9·4 | 9·6 |
| Brunelin | 2014 | hamd-17 | 50 | 26·1 | 3·9 | 15·4 | 8·3 | 51 | 25·8 | 3·4 | 14·5 | 9·9 |

**Supplementary Table 4.** Raw data used in response analysis

| **Author** |  | **Year** | **Active**  **N** | **Number of Active Responders** | **Control N** | **Number of Control Responders** |
| --- | --- | --- | --- | --- | --- | --- |
| Chen |  | 2022 | 49 | 31 | 48 | 14 |
| Rossini SSRI |  | 2005 | 30 | 22 | 30 | 22 |
| Wang |  | 2017 | 22 | 20 | 21 | 18 |
| Ahmadpanah |  | 2023 | 18 | 15 | 17 | 8 |
| Huang |  | 2012 | 28 | 13 | 28 | 10 |
| Wang |  | 2023 | 60 | 57 | 60 | 48 |
| Rossini SNRI |  | 2005 | 15 | 14 | 14 | 10 |
| Ullrich |  | 2012 | 22 | 4 | 21 | 0 |
| Herwig |  | 2018 | 62 | 19 | 65 | 20 |
| Brunelin |  | 2014 | 50 | 27 | 51 | 31 |

**Supplementary Table 5.** Raw data used in remission analysis

| **Author** |  | **Year** | **Active**  **N** | **Number of Active Remitters** | **Control N** | **Number of Control Remitters** |
| --- | --- | --- | --- | --- | --- | --- |
| Chen |  | 2022 | 49 | 18 | 48 | 6 |
| Rossini SSRI |  | 2005 | 30 | 21 | 30 | 17 |
| Wang |  | 2017 | 22 | 19 | 21 | 16 |
| Huang |  | 2012 | 28 | 11 | 28 | 8 |
| Wang |  | 2023 | 60 | 23 | 60 | 7 |
| Rossini SNRI |  | 2005 | 15 | 12 | 14 | 7 |
| Ullrich |  | 2012 | 22 | 0 | 21 | 0 |
| Herwig |  | 2018 | 62 | 6 | 65 | 10 |
| Brunelin |  | 2014 | 50 | 14 | 51 | 22 |

**Supplementary Table 6.** Safety and tolerability of studies included in the meta-analysis

| **Study** | **Medication Class** | **Serious Adverse Events** | **Other Adverse Events** | **Safety / Tolerability (drop out numbers, side effects)** |
| --- | --- | --- | --- | --- |
| Chen et al., 2022 | SSRI | ·· | ·· | Not reported |
| Rossini et al., 2005 SSRIs | SSRI | rTMS: 0·0%  Sham: 0·0 % | rTMS: 0·0%  Sham: 3·3% | 1 patient in the sham + escitalopram group dropped out due to intolerable agitation and gastric symptoms, headache, and cervical pain. 7 patients dropped out for other reasons (lack of improvement, consent withdrawal, leaving for holiday, missed visits). No patients developed psychotic symptoms |
| [Wang et al., 2017](https://www.sciencedirect.com/science/article/pii/S0165178116312999?via%3Dihub) | SSRI | rTMS: 0·0%  Sham: 0·0 % | rTMS:23 %  Sham: 33% | 12 participants reported headache or scalp pain (5 in the rTMS group and 7 in the sham group; 22·7% and 33·3%, respectively) at the end of the 4th week.  At the end of the 8th week 2 in the rTMS group and 1 in the sham group |
| [Dai et al., 2020](https://journals.lww.com/md-journal/Fulltext/2020/08070/The_therapeutic_effect_of_repetitive_transcranial.25.aspx) | SSRI | rTMS: 0·0%  Sham: 0·0 % | rTMS: 8·1%  Sham: 4·8% | Five participants in the rTMS group reported dizziness, nausea, and chest tightness, 2 of which dropped out. 3 patients in the sham group had nausea, mouth dryness, constipation, and headache. 4 participants in the rTMS group reported mild headaches. |
| Guan et al., 2021 | SSRI | ·· | ·· | Not reported |
| García-Toro et al., 2001 | SSRI | rTMS: 0·0%  Sham: 0·0% | rTMS: 27%  Sham: 0·0% | 6 patients dropped out (3 sham and 3 active rTMS). 1 due to intolerance to sertraline, 1 for “fear of brain damage”. 4 patients were lost in 2 weeks of follow-up after rTMS (3 for unacceptable changes in medication, 1 for “personal reasons”). 3/11 patients in rTMS group reported muscle tension headaches |
| [Ahmadpanah et al., 2023](https://www.mdpi.com/2077-0383/12/7/2525) | SSRI | ·· | ·· | 3 patients lost to follow up in sham and 2 in active condition |
| [Bretlau et al., 2008](https://www.thieme-connect.com/products/ejournals/html/10.1055/s-2007-993210?casa_token=p7lalJzaIRAAAAAA:ElE1DukCef7ZXiWDd6B7NWs2vNg6r11AYVP2wQfkG-CbC7UkbrPcUHdxfPOiYlludexbdy-6kVoYXVbVBA) | SSRI | rTMS: 0·0%  Sham: 0·0% | ·· | During the 9-week post-TMS phase with escitalopram, 5 patients from the sham and 1 patient from the rTMS group dropped out |
| Huang et al., 2012 | SSRI | rTMS: 0·0%  Sham: 0·0% | rTMS: 29%  Sham: 0·0% | 2 patients in the active group dropped out due to pain at the site of stimulation, and 2 in the sham group were unable to tolerate the sound of the stimulation. In the active group, 3 patients reported cephalgia and 3 fatigue. |
| Wang et al., 2023 | SSRI | rTMS: 0·0%  Sham: 0·0% | .. | There were no dropouts or serious adverse events in either group. |
| [Rossini et al., 2005 SNRIs](https://www.psychiatrist.com/jcp/depression/does-rtms-hasten-response-escitalopram-sertraline/) | SNRI | rTMS: 0·0%  Sham: 0·0 % | rTMS: 6·7%  Sham: 7·1% | 1 patient in active TMS + venlafaxine and 1 patient in sham + venlafaxine dropped out due to intolerable agitation and gastric symptoms, headache, and cervical pain. 7 patients dropped out for other reasons (lack of improvement, consent withdrawal, leaving for holiday, missed visits). No patients developed psychotic symptoms |
| [Ullrich et al., 2012](https://karger.com/nps/article/66/3/141/233488/Ultra-High-Frequency-Left-Prefrontal-Transcranial) | SNRI | rTMS: 0·0%  Sham: 0·0 % | rTMS: 0·0%  Sham: 0·0 % | All patients completed the study. No adverse events were reported by the patients. |
| [Brunelin et al., 2014](https://www.sciencedirect.com/science/article/pii/S1935861X14002691?casa_token=fNAOQ4A-LpwAAAAA:eQRqa0p8HYfWwaCf58QTdS1h9Eqq8CY6eKbBhFo4IBv38_fhyCsMveybHctSWfbdGrcTjYo_7qJ_) | SNRI | rTMS: 14%  Sham: 3·9% | ·· | The discontinuation rate for the sham group was 23% and 22% for the active group. There was no difference in the severity of adverse events (7 in the sham and 2 in the active group). 4 patients were hospitalized |
| [Herwig et al., 2018](https://www.cambridge.org/core/journals/the-british-journal-of-psychiatry/article/antidepressant-effects-of-augmentative-transcranial-magnetic-stimulation/65C63C38F7720FAA0D3AD422BA267C3C) | SNRI | rTMS: 0·0%  Sham: 0·0 % | rTMS: 8·1%  Sham: 6·2% | Patients in both groups reported headaches and painful local sensations. A patient in the sham groups reported dizziness and 1 patient in the rTMS group reported nausea. No patients reported any serious adverse events. |


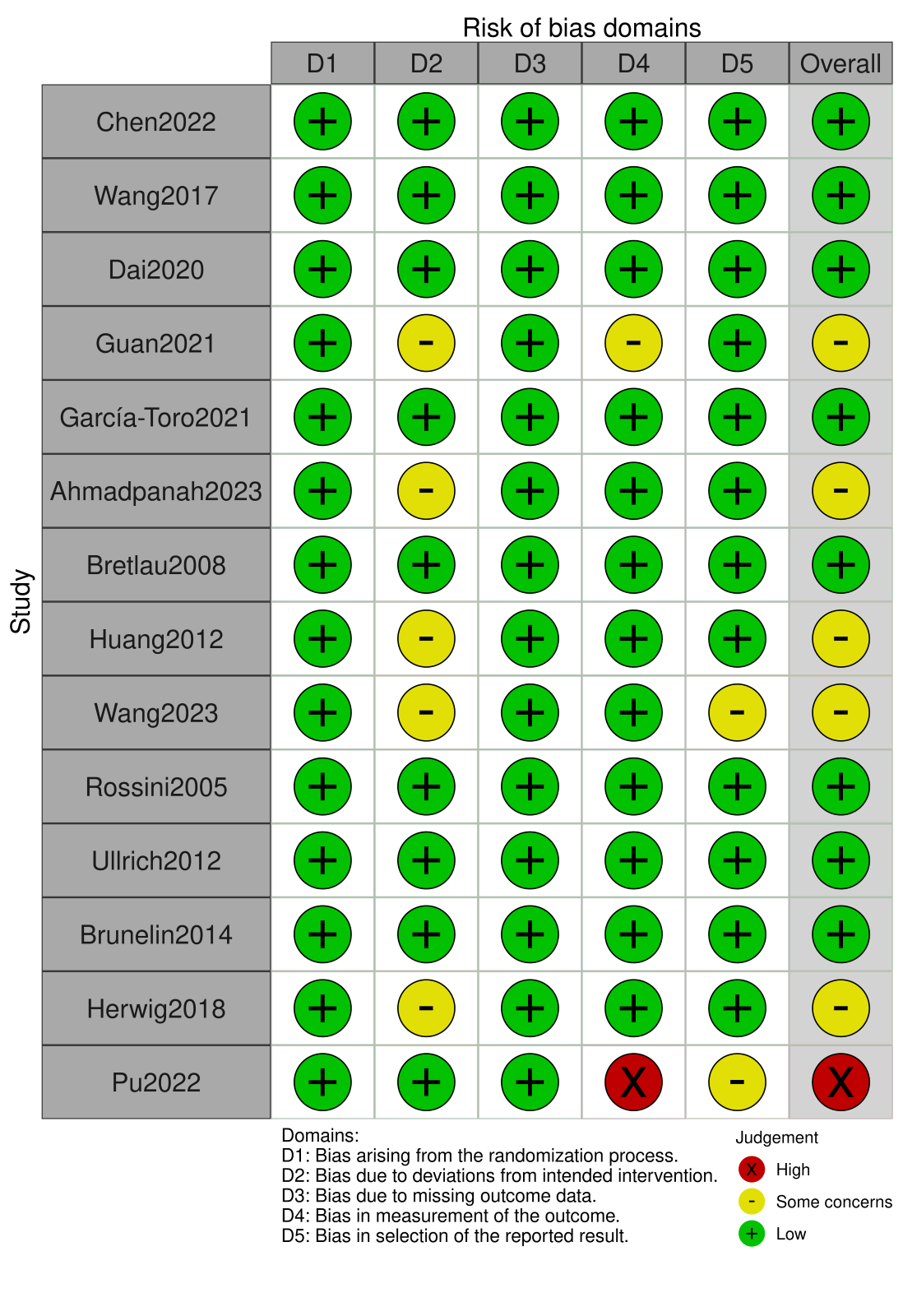


**Supplementary Fig. 1:** Study Level Cochrane Risk of Bias Analysis Results


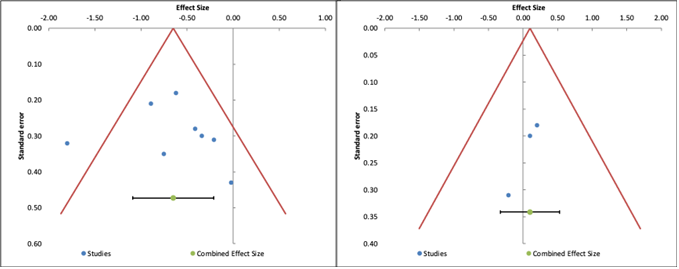
**Supplementary Fig. 2:** Funnel Plot of Primary Outcome Variable ∆ in Depression Severity

In the above plot the red line shows 95% confidence intervals, the blue points plot each study’s Standardized Mean Difference (SMD) and standard error, the green point represents the combined effect size with 95% confidence interval bars extending on either side. The first panel shows the funnel plot of the SSRI group with an outlier to the far left (Huang, 2012). The second panel shows a funnel plot for the SNRI group.


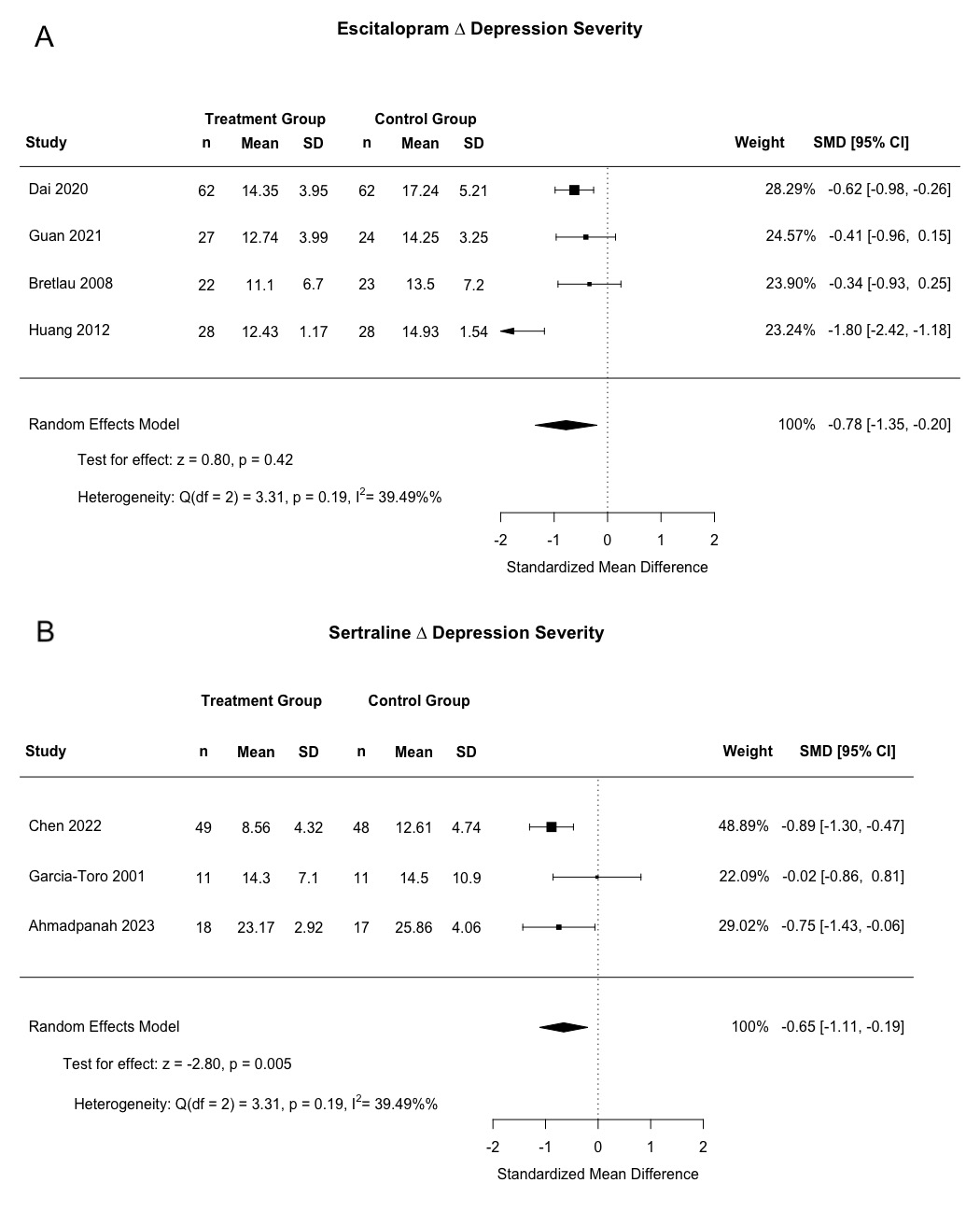


**Supplementary Fig. 3:** Subgroup Analysis of Effect of SSRI Type on ∆ in Depression Severity Panels A and B shows forest plots of the Standardized Mean Difference (SMD) of depression severity in treatment vs control groups for escitalopram (A) and sertraline (B). Horizontal bars show 95% confidence intervals, with studies closer to the dashed vertical line having no effect on depression severity.


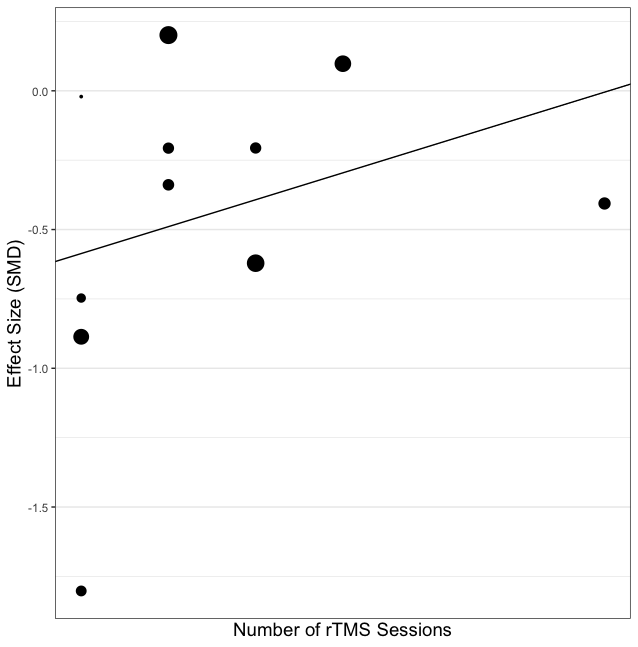


**Supplementary Fig. 4:** Meta-Regression of Number of rTMS Treatment Sessions. In the above meta-regression, the x-axis represents the moderator variable, number of rTMS treatment sessions. The y-axis shows the effect size of the primary outcome of change in depression severity measured in standardized mean differences (SMD). The plot points are weighted so that studies with a lower standard error have a higher weight. The regression analysis was non-significant (p=0.32) and the model did not explain a large portion of heterogeneity (I^2^=78.57%).


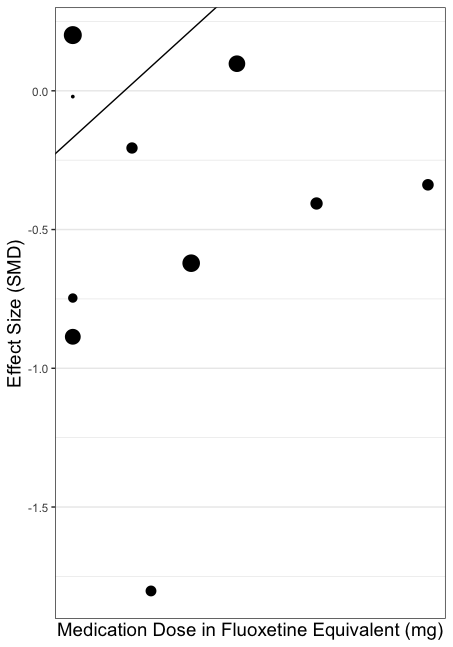


**Supplementary Fig. 5:** Meta-Regression of Medication Dose. In the above meta-regression, the x-axis represents the moderator variable, dose of medication in fluoxetine equivalent (mg). The y-axis shows the effect size of the primary outcome of change in depression severity measured in standardized mean differences (SMD). The plot points are weighted so that studies with a lower standard error have a higher weight. The regression analysis was non-significant (p=0.81) and the model did not explain a large portion of heterogeneity (I^2^=82.85%).


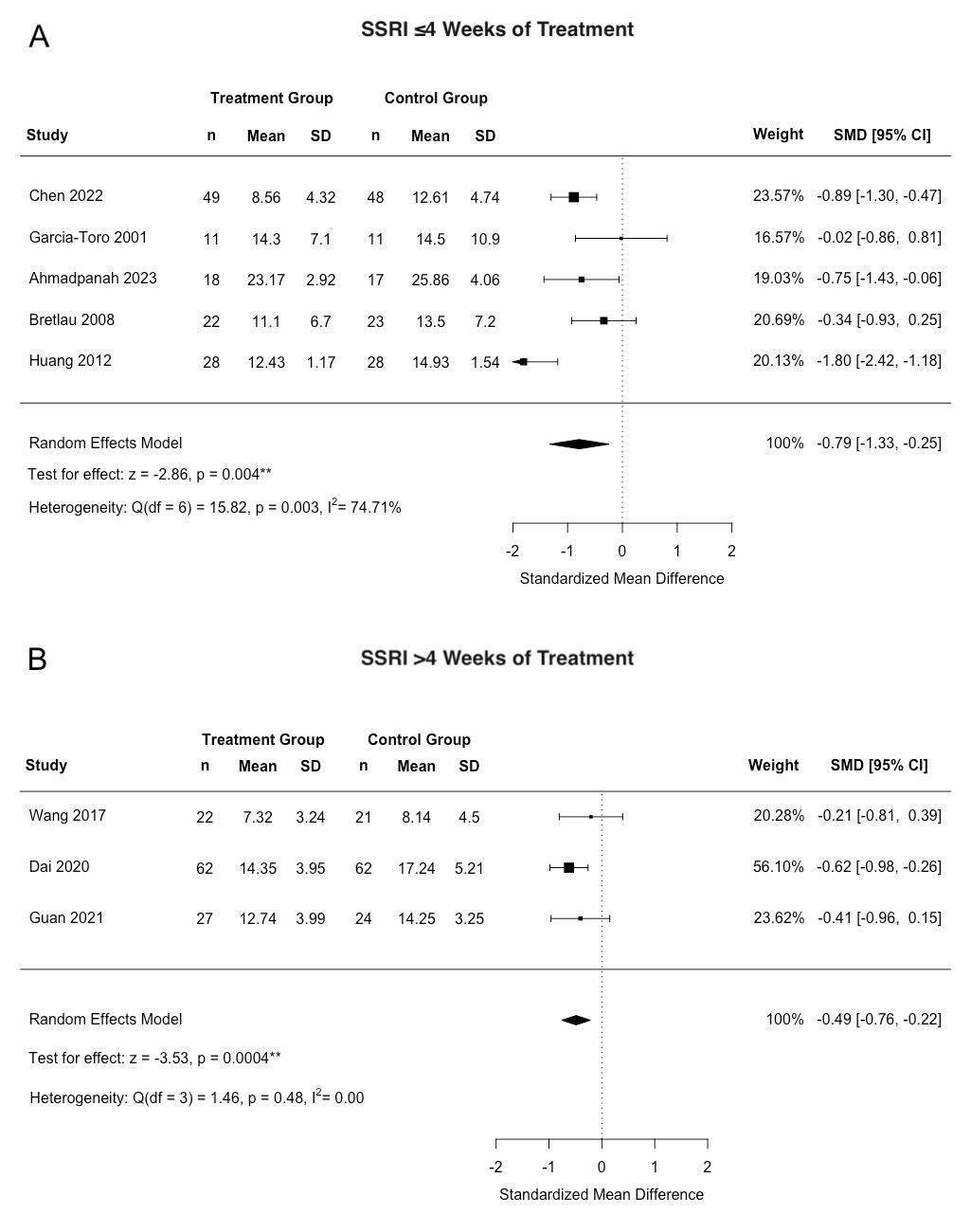


**Supplementary Fig. 6**: Subgroup analysis of effect of treatment duration on treatment outcomes in SSRI studies. Panels A and B show forest plots of the Standardized Mean Difference (SMD) of change in depression severity in treatment vs control groups for all SSRI studies assessing (A) ≤4 weeks of treatment versus (B) >4 weeks of treatment. Horizontal bars show 95% confidence intervals, with studies closer to the dashed vertical line having no effect on depression severity. Analyses of response/remission outcomes were not included in this sub-group analysis due to small sample sizes for each group as not all studies reported these outcomes.


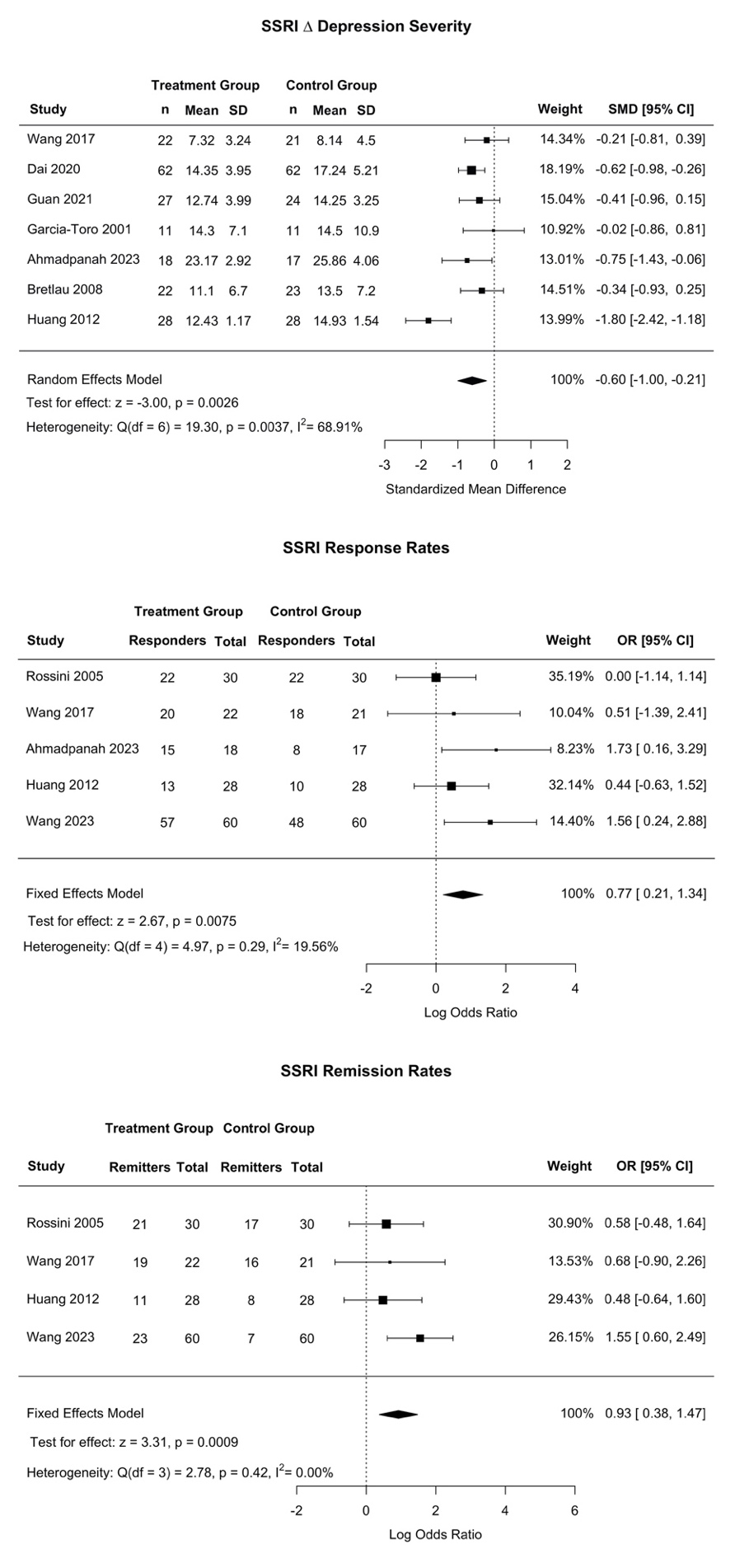


**Supplementary Fig 7.** Sensitivity analysis for SSRI results with the Chen et al. study removed, which included patients with first episode major depression.


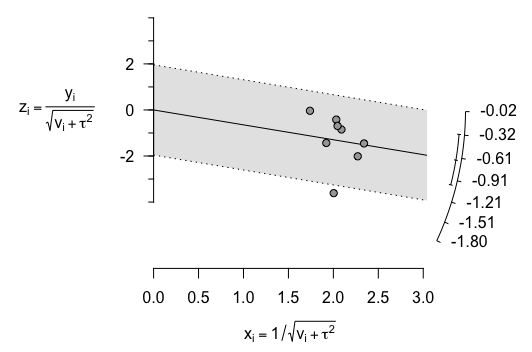


**Supplementary Fig 8.** Radial Plot of Primary Outcome Variable ∆ in Depression Severity in SSRI Studies.

In the above plot, the horizontal axis represents the inverse of the standard error, and the vertical axis is the standardized estimates or z-statistic.


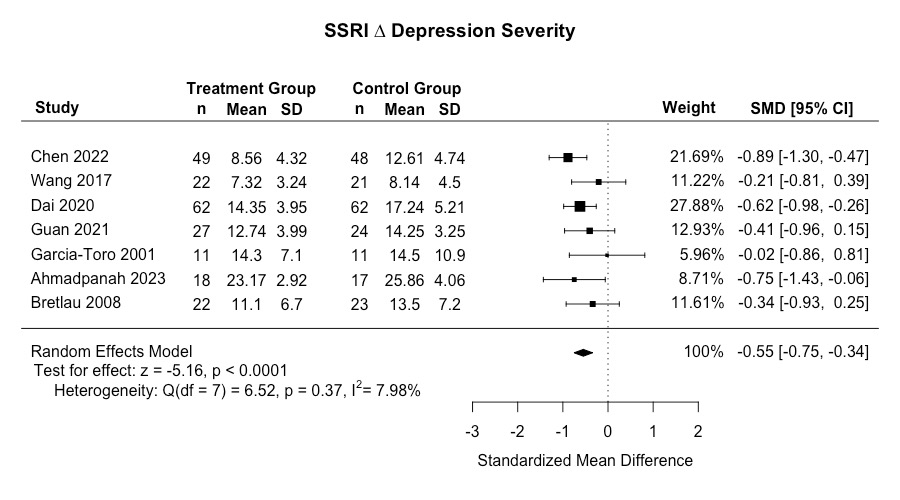
**Supplementary Fig. 9:** Sensitivity analysis of Primary Outcome Variable ∆ in Depression Severity with the Huang et al. Study Removed, which was found to be an outlier on radial plot analysis.


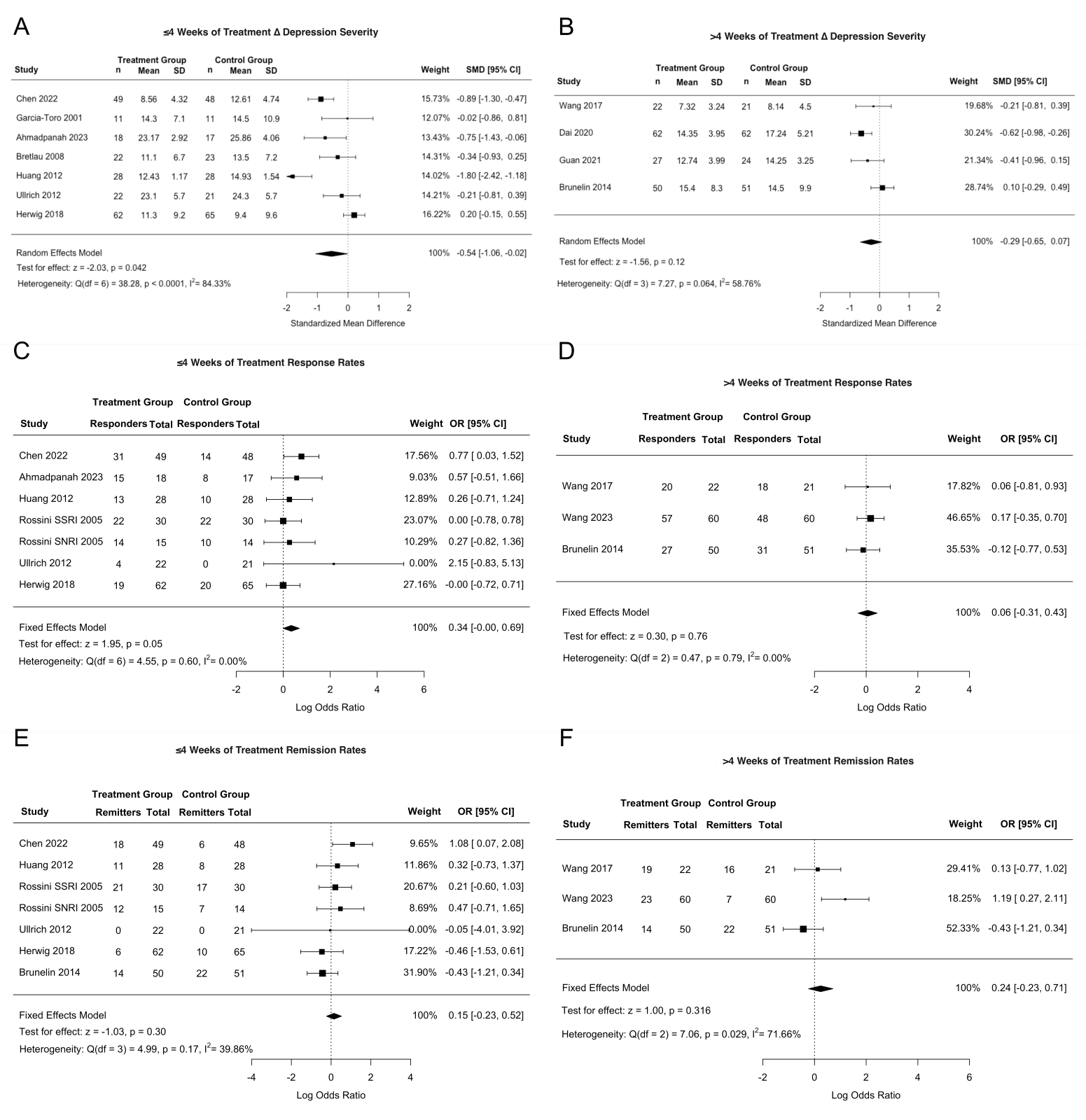


**Supplementary Fig. 10:** Subgroup analysis of effect of treatment duration on treatment outcomes for all studies (i.e. pooled SNRI/SSRI studies). Panels A and B show forest plots of the Standardized Mean Difference (SMD) of change in depression severity in treatment vs control groups for studies assessing (A) ≤4 weeks of treatment and (B) >4 weeks of treatment. Panels C to F show Odds Ratios (OR) of response (C-D) and remission rates (E-F) of treatment and control groups for studies assessing acute and long-term effects. Horizontal bars show 95% confidence intervals, with studies closer to the dashed vertical line having no effect on depression severity.
