# Supplementary material for: Antidepressant class and concurrent rTMS outcomes in major depressive disorder: a systematic review and meta-analysis
Source: eClinicalMedicine. 2024 Jul 27;75:102760. doi: 10.1016/j.eclinm.2024.102760 (PMC11338161; doi:10.1016/j.eclinm.2024.102760)
Supplement: Supplementary_Information_Methods [file mmc2.docx]

**Supplementary Methods**

**Supplementary Methods:**

**Supplementary Methods 1.** Full search strategy and terms for Embase and Medline

**Supplementary Methods 2.** Full search strategy and terms for APA psychINFO

**Supplementary Methods 3.** Full search strategy and terms for Cochrane Search

**Methods 1.** Full search strategy and terms for Embase and Medline

(TMS or rTMS or dTMS or iTBS or TBS or cTBS):ti,ab,kw OR (transcranial magnetic NEXT (stimulation*)):ti,ab,kw OR (theta-burst NEXT (stimulation*)):ti,ab,kw AND ("theta burst stimulation"):ti,ab,kw

| 1. | exp Depression/ |
| --- | --- |
| 2. | exp depressive disorder, major/ or exp depressive disorder, treatment-resistant/ or exp vascular depression/ |
| 3. | (depress* or MDD or TRD).tw,kf. |
| 4. | exp Transcranial Magnetic Stimulation/ |
| 5. | (transcranial magnetic stimulation* or TMS or rTMS or dTMS or iTBS or TBS or theta burst stimulation*).tw,kf. |
| 6. | exp Psychotropic Drugs/ |
| 7. | exp Drug Therapy/ |
| 8. | exp Selective Serotonin Reuptake Inhibitors/ |
| 9. | exp antidepressive agents/ or exp antidepressive agents, tricyclic/ |
| 10. | exp Monoamine Oxidase Inhibitors/ |
| 11. | exp Benzodiazepines/ |
| 12. | exp Antipsychotic Agents/ |
| 13. | (antidepressant drug* or antidepressant medication* or antidepressive agent* or psychoactive agent* or psychoactive drug* or psychopharmaceutical* or psychotropic drug* or serotonin reuptake inhibitor* or SSRI or norepinephrine reuptake inhibitor* or SNRI* or Bupropion or Desvenlafaxine or Duloxetine or Levomilnacipran or Milnacipran or Venlafaxine or Amoxapine or Citalopram or Clomipramine or Escitalopram or Fluoxetine or Olanzapine or Paroxetine or Sertraline or Trazodone or Vilazodone or Vortioxetine or Clorgyline or Iproniazid or Isocarboxazid or Moclobemide or Phenelzine or Tranylcypromine or Amitriptyline or Amoxapine or Desipramine or Dothiepin or Dosulepin or Doxepin or Imipramine or Iprindole or Lofepramine or Opipramol or Protriptyline or Trimipramine or Ketamine or mirtazapine or fluvoxamine or esketamine or Nefazodone or reboxetine).tw,kf. |
| 14. | ((antidepress* or anti depress* or psychoactive or psycho active or psychotropic) adj2 (drug* or medicat* or agent* or prescription*)).tw,kf. |
| 15. | 1 or 2 or 3 |
| 16. | 4 or 5 |
| 17. | 6 or 7 or 8 or 9 or 10 or 11 or 12 or 13 or 14 |
| 18. | 15 and 16 and 17 |

**Methods 2.** Full search strategy and terms for APA psychINFO

| 1. | exp Transcranial Magnetic Stimulation/ |
| --- | --- |
| 2. | (transcranial magnetic stimulation* or TMS or rTMS or dTMS or iTBS or TBS or theta burst stimulation*).tw. |
| 3. | exp Major Depression/ |
| 4. | (depress* or MDD or treatment resistant depress*).tw. |
| 5. | exp Psychotropic Drugs/ |
| 6. | exp antidepressant drugs/ |
| 7. | exp Serotonin Reuptake Inhibitors/ |
| 8. | exp Psychopharmacology/ |
| 9. | exp Neuroleptic Drugs/ |
| 10. | exp monoamine oxidase inhibitors/ or exp tricyclic antidepressant drugs/ |
| 11. | exp Benzodiazepines/ |
| 12. | (antidepressant drug* or antidepressant medication* or antidepressive agent* or psychoactive agent* or psychoactive drug* or psychopharmaceutical* or psychotropic drug* or serotonin reuptake inhibitor* or SSRI or norepinephrine reuptake inhibitor* or SNRI* or Bupropion or Desvenlafaxine or Duloxetine or Levomilnacipran or Milnacipran or Venlafaxine or Amoxapine or Citalopram or Clomipramine or Escitalopram or Fluoxetine or Olanzapine or Paroxetine or Sertraline or Trazodone or Vilazodone or Vortioxetine or Clorgyline or Iproniazid or Isocarboxazid or Moclobemide or Phenelzine or Tranylcypromine or Amitriptyline or Amoxapine or Desipramine or Dothiepin or Dosulepin or Doxepin or Imipramine or Iprindole or Lofepramine or Opipramol or Protriptyline or Trimipramine or Ketamine or mirtazapine or fluvoxamine or esketamine or Nefazodone or reboxetine).tw. |
| 13. | ((antidepress* or anti depress* or psychoactive or psycho active or psychotropic) adj2 (drug* or medicat* or agent* or prescription*)).tw. |
| 14. | 1 or 2 |
| 15. | 3 or 4 |
| 16. | 5 or 6 or 7 or 8 or 9 or 10 or 11 or 12 or 13 |
| 17. | 14 and 15 and 16 |

**Methods 3.** Full search strategy and terms for Cochrane Search

| 1 | MeSH descriptor: [Depression] explode all trees |
| --- | --- |
| 2 | MeSH descriptor: [Depressive Disorder, Major] explode all trees |
| 3 | (depress*):ti,ab,kw OR ("treatment resistant depression"):ti,ab,kw OR ("major depression disorder"):ti,ab,kw OR (TRD):ti,ab,kw OR (MDD):ti,ab,kw (Word variations have been searched) |
| 4 | MeSH descriptor: [Transcranial Magnetic Stimulation] explode all trees |
| 5 | (TMS OR rTMS OR dTMS OR iTBS OR TBS OR cTBS):ti,ab,kw OR (transcranial magnetic NEXT (stimulation*)):ti,ab,kw OR (theta-burst NEXT (stimulation*)):ti,ab,kw OR ("theta burst stimulation"):ti,ab,kw (Word variations have been searched) |
| 6 | MeSH descriptor: [Psychotropic Drugs] explode all trees |
| 7 | MeSH descriptor: [Drug Therapy] explode all trees |
| 8 | MeSH descriptor: [Neurotransmitter Agents] explode all trees |
| 9 | MeSH descriptor: [Enzyme Inhibitors] explode all trees |
| 10 | MeSH descriptor: [Benzodiazepines] explode all trees |
| 11 | MeSH descriptor: [Tranquilizing Agents] explode all trees |
| 12 | (antidepress* NEXT (drug* OR medication* OR agent*)):ti,ab,kw OR (psychoactive NEXT (drug* OR medication* OR agent*)):ti,ab,kw OR (psychotropic NEXT (drug* OR medication* OR agent*)):ti,ab,kw OR ("psychopharmaceutical"):ti,ab,kw OR (serotonin reuptake inhibitor* OR SSRI OR norepinephrine reuptake inhibitor* OR SNRI* OR tricyclic antidepressant* OR TCA* OR monoamine oxidase inhibitor* OR MAOI* OR atypical antidepressants OR benzodiazepine* OR Bupropion OR Desvenlafaxine OR Duloxetine OR Levomilnacipran OR Milnacipran or Venlafaxine OR Amoxapine OR Citalopram OR Clomipramine OR Escitalopram OR Fluoxetine OR Olanzapine OR Paroxetine OR Sertraline OR Trazodone OR Vilazodone OR Vortioxetine OR Clorgyline OR Iproniazid OR Isocarboxazid OR Moclobemide OR Phenelzine OR Tranylcypromine OR Amitriptyline OR Amoxapine OR Desipramine OR Dothiepin OR Dosulepin OR Doxepin OR Imipramine OR Iprindole OR Lofepramine OR Opipramol OR Protriptyline OR Trimipramine OR Ketamine OR mirtazapine OR fluvoxamine OR esketamine OR Nefazodone OR reboxetine):ti,ab,kw (Word variations have been searched) |
| 13 | #1 OR #2 OR #3 |
| 14 | #4 OR #5 |
| 15 | #6 OR #7 OR #8 OR #9 OR #10 OR #11 OR #12 |
| 16 | #13 AND #14 AND #15 |
